# Novel γ-sarcoglycan interactors in murine muscle membranes

**DOI:** 10.1186/s13395-021-00285-2

**Published:** 2022-01-22

**Authors:** Tara C. Smith, Georgios Vasilakos, Scott A. Shaffer, Jason M. Puglise, Chih-Hsuan Chou, Elisabeth R. Barton, Elizabeth J. Luna

**Affiliations:** 1 Department of Radiology, Division of Cell Biology & Imaging, University of Massachusetts Medical School, Worcester, MA USA; 2 Applied Physiology & Kinesiology, College of Health & Human Performance, University of Florida, Gainesville, FL USA; 3 Biochemistry and Molecular Pharmacology, University of Massachusetts Medical School, Worcester, MA USA; 4 Mass Spectrometry Facility, University of Massachusetts Medical School, Shrewsbury, MA USA

**Keywords:** Limb girdle muscular dystrophy, Skeletal muscle, Sarcolemma, Archvillin, Svil, PP1β/δ, Sarcoglycans, NKCC1

## Abstract

**Background:**

The sarcoglycan complex (SC) is part of a network that links the striated muscle cytoskeleton to the basal lamina across the sarcolemma. The SC coordinates changes in phosphorylation and Ca^++^-flux during mechanical deformation, and these processes are disrupted with loss-of-function mutations in gamma-sarcoglycan (Sgcg) that cause Limb girdle muscular dystrophy 2C/R5.

**Methods:**

To gain insight into how the SC mediates mechano-signaling in muscle, we utilized LC-MS/MS proteomics of SC-associated proteins in immunoprecipitates from enriched sarcolemmal fractions. Criteria for inclusion were co-immunoprecipitation with anti-Sgcg from C57BL/6 control muscle and under-representation in parallel experiments with Sgcg-null muscle and with non-specific IgG. Validation of interaction was performed in co-expression experiments in human RH30 rhabdomyosarcoma cells.

**Results:**

We identified 19 candidates as direct or indirect interactors for Sgcg, including the other 3 SC proteins. Novel potential interactors included protein-phosphatase-1-catalytic-subunit-beta (Ppp1cb, PP1b) and Na^+^-K^+^-Cl^−^-co-transporter NKCC1 (SLC12A2). NKCC1 co-localized with Sgcg after co-expression in human RH30 rhabdomyosarcoma cells, and its cytosolic domains depleted Sgcg from cell lysates upon immunoprecipitation and co-localized with Sgcg after detergent permeabilization. NKCC1 localized in proximity to the dystrophin complex at costameres in vivo. Bumetanide inhibition of NKCC1 cotransporter activity in isolated muscles reduced SC-dependent, strain-induced increases in phosphorylation of extracellular signal-regulated kinases 1 and 2 (ERK1/2). In silico analysis suggests that candidate SC interactors may cross-talk with survival signaling pathways, including p53, estrogen receptor, and TRIM25.

**Conclusions:**

Results support that NKCC1 is a new SC-associated signaling protein. Moreover, the identities of other candidate SC interactors suggest ways by which the SC and NKCC1, along with other Sgcg interactors such as the membrane-cytoskeleton linker archvillin, may regulate kinase- and Ca^++^-mediated survival signaling in skeletal muscle.

**Supplementary Information:**

The online version contains supplementary material available at 10.1186/s13395-021-00285-2.

## Background

The dystrophin glycoprotein complex (DGC) is a major sarcolemmal complex that tethers the extracellular matrix (ECM) to the intracellular actin cytoskeleton [1, 2]. Together with the integrin-associated focal adhesion complex [3, 4], the DGC stabilizes the membrane, senses mechanical stress, and transmits “outside-in” information to the nucleus via signaling proteins, including phosphorylated signaling kinases [5, 6]. The dominant DGC connection to actin is via dystrophin, which links the actin cytoskeleton to dystroglycan, and in turn binds laminin in the ECM [1, 2]. The sarcoglycan complex (SC) is a sub-complex of the DGC, comprised of α-, β-, δ-, and γ-sarcoglycans (Sgca, Sgcb, Sgcd, Sgcg) in skeletal muscle [7]. These proteins form a heterotetramer, each having a short intracellular domain, a single membrane-spanning α-helix, and a large extracellular domain. Sarcolemmal DGC proteins are concentrated at costameres, which are associated with the mouths of T-tubules and contain a multitude of signaling proteins [2, 8–13].

Deficiencies in many DGC proteins are causal for muscular dystrophies in humans and characterized by disarranged costameres, progressive muscle loss, and shortened lifespan [14, 15]. Loss of any SC protein in humans or mice leads to the significant reduction of the entire sub-complex and to a limb girdle muscular dystrophy (LGMD) [15]. LGMDs are characterized by severe pathology, including elevated serum creatine kinase levels, cycles of muscle degeneration and regeneration, and extensive fibrosis, yet there is variable mechanical fragility [16, 17]. Mice lacking a functional SC exhibit altered signaling through the mitogen-activated protein kinases (MAPK), including extracellular signal-regulated kinases 1 and 2 (ERK1/2) [18–21] and p38 [22], as well as increased levels of intracellular calcium ions [23] during eccentric contractions (ECC) of skeletal muscle. Although the signaling pathways have not been elucidated, Sgcg tyrosine-6 is required for SC-dependent changes in the level of phosphorylated ERK1/2 [18, 20, 24], and mutation of Sgcg-Y6 is associated with severe auto-recessive muscular dystrophy (reviewed in [5]). Yeast two-hybrid interactors have been described for the cytosolic N-termini of the four SC proteins [25, 26], but proteomic analyses have been confounded by the relative scarcity of these proteins in muscle.

One candidate interaction partner is archvillin [27], a membrane-associated cytosolic protein that binds directly to dystrophin, interacts with Sgcg in yeast two-hybrid assays, and is required for the structural integrity of human muscles [26, 28]. Archvillin is implicated in ERK1/2-regulated survival signaling in muscle [29], as is Sgcg. Smooth muscle archvillin scaffolds B-Raf and ERK1/2 to promote ERK signaling [30]. In skeletal muscle, Sgcg regulates archvillin association with P-ERK1/2 following ECC, which is ablated in the absence of Sgcg or dystrophin, and leads to heightened basal P-ERK1/2, increased nuclear P-ERK1/2 after active mechanical perturbation, and an uncoupled signaling response to passive stretch [26]. A nonmuscle isoform (supervillin, SV1), which is encoded by the same Svil gene as archvillin [27, 31], regulates focal adhesion formation and cell survival [32–34].

While it is clear that Sgcg and archvillin coordinate to modulate mechanical signaling in muscle, it leaves open the possibility for additional direct or indirect protein interactors to participate in these actions. To look for such Sgcg-associated proteins, we developed a medium-throughput method for carrying out co-IPs of Sgcg and other sarcolemmal proteins. We identified 16 novel candidate Sgcg interactors by LC MS-MS analyses of co-IPs from single mouse muscles with and without the SG complex. We confirmed direct binding of the Sgcg N-terminus with the archvillin C-terminus and demonstrated an interaction of SC with N- and C-terminal cytoplasmic domains of the Na^+^-K^+^-2Cl^−^-cotransporter (NKCC1). We also report for the first time that NKCC1 immunofluorescence overlaps with that of costameric dystrophin at the sarcolemma and that NKCC1 cotransporter activity is required for the ECC-induced increases in activated ERK seen in *Sgcg*^*−/−*^ mouse muscle. These results are the first to localize NKCC1 at or near muscle costameres and to implicate this channel in SG-mediated signaling.

## Methods

### Affinity-purified rabbit polyclonal anti-γ-sarcoglycan and anti-archvillin

Rabbit polyclonal antibodies specific for extracellular residues 72–290 in murine γ-sarcoglycan (Sgcg, NCBI reference sequence NP_036022.1) and for murine archvillin (mAV, Swiss Protein Q8K463.1) amino acids 121–568 were generated against bacterially expressed proteins at Cocalico Biologicals, Inc. (Stevens, PA). Affinity purification employed columns containing the corresponding GST-tagged proteins and protocols detailed previously for high-avidity antibodies against human supervillin [35]. After a series of stringent washes, high-avidity antibodies were eluted from a column with covalently bound immunogen using 4.5 M MgCl_2_, 72.5 mM Tris-HCl pH 6.0–7.0. PCR templates were a murine Sgcg plasmid from Dr. Elizabeth McNally [36] and an EGFP-tagged mAV plasmid [27], respectively. PCR primers included the underlined sites for directed restriction cloning, as shown in Additional file 1, Supplementary Table S1. PCR products were generated with Pfu Turbo DNA polymerase (Agilent Technologies, Santa Clara, CA) according to the manufacturer’s directions, gel purified, cloned into TOPO-pCR2.1 vector (ThermoFisher Scientific, Waltham, MA), and verified by DNA sequencing. Coding sequences for Sgcg and mAV were recovered from doubly digested vectors and ligated into identically digested pGEX-6P-1 (Sigma-Aldrich, St. Louis, MO). Soluble GST-mAV was isolated from Rosetta 2(DE3)pLysS chemically competent bacteria (EMD-Millipore-Sigma, Burlington, MA) induced overnight with 0.2 mM isopropyl b-D-thiogalactopyranoside and purified on glutathione-Sepharose™ (Sigma-Aldrich). The ~ 26-kDa mAV immunogen was generated by cleavage with PreScission Protease (Sigma-Aldrich), dialyzed to remove residual glutathione, and purified by removing GST with a second glutathione-Sepharose column, followed by electrophoresis on a 15% acrylamide SDS-gel [37]. Because GST-Sgcg was incorporated into inclusion bodies and was poorly cleaved after urea renaturation, His-tagged Sgcg was generated by cloning the *BamHI* and *EcoRI* DNA fragment into doubly cut pET-30a vector (Sigma-Aldrich). Urea-solubilized His-Sgcg was purified on Ni-NTA agarose columns (Qiagen, Germantown, MD), as described [38], eluting with 50-100 mM histidine in a step gradient of 10–250 mM histidine in column buffer (7 M urea, 5 mM glycine, 50 mM Tris-HCl, pH 8.0, 0.3 M NaCl, 0.5 mM DTT, 1 mM PMSF). The ~ 29-kDa His-Sgcg remained soluble after dialysis against 1 mM DTT, PBS, pH 7.4, and was used as immunogen without further purification. Rabbits were boosted until high titers were observed on immunoblots, and each antibody was affinity purified with the corresponding GST-tagged protein covalently bound to CNBr-activated Sepharose (Sigma-Aldrich, C9142) [35]. After affinity purification, the rabbit anti-mAV antibody was used at 1:5000–1:10000 for immunoblots (IB), and the rabbit anti-Sgcg was used at 1:5000 for IB and at 1:200 for immunofluorescence microscopy (IF).

### Commercial antibodies

Dilutions refer to those used for IB, IF, and immunoprecipitations (IP). Murine monoclonal anti-HA-Tag (6E2, #2367; IB: 1:1500, IF: 1:200) and anti-DYKDDDDK/Flag epitope (9A3, #8146; IB: 1:1000, IF: 1:200) antibodies were obtained from Cell Signaling Technologies (Beverly, MA). Murine monoclonal hybridoma supernatants were obtained from the Developmental Studies Hybridoma Bank (Iowa City, IA) to stain dystrophin (MANDRA1, 7A10, IB: 1:1000; MANDYS1, 3B7, IF 1:10) [39], α-sarcoglycan (IVD3(1)A9; IB 1:50), β-dystroglycan (MANDAG1 (7A11); IB 1:200), contractile myosins II (A4.1025; IB 1:100) [40, 41], troponin T (JLT12; IB 1:300) and SERCA1 (CaF2-5D2; IB 1:70). Rabbit monoclonal anti-HA (C29F4, #3724; IB: 1:1000, IP: 18 μl/60 μl of Dynabeads) and affinity-purified rabbit polyclonal anti-DYKDDDDK (#2368; IB: 1:1000, IP: 18 μl per 60 μl of Dynabeads) antibodies were from Cell Signaling Technologies. Rabbit monoclonal anti-PP1β was from Abcam (#ab53315, Cambridge, MA, USA; IB: 1:1000, IF: 1:100). Affinity-purified rabbit polyclonal antibodies against MYPT2 (#13366-1-AP; IB: 1:1000), NKCC1 (#13884-1-AP; IB: 1:1000, IF: 1:500) and Sgcg (#18102-1-AP; IB: 1:1000, IF: 1:100) were purchased from Proteintech Group Inc. (Rosemont, IL). Other rabbit polyclonal antibodies were anti-PP1β (#LS-C482256, Life Span BioSciences Inc., Seattle, WA; IB: 1:500), anti-NKCC1 (#ANT-071, Alomone Labs, Radassah Einkerem, Jerusalem, Israel; IB: 1:500, IF: 1:50), and anti-ERK1/2 (#ABS44, EMD Millipore Sigma; IB: 1:1000). Isotype-specific control antibodies were rabbit IgG (EMD-Millipore #12-370) and mouse IgG2a (#401502, BioLegend, San Diego, CA). For chemiluminescence detection in IB, we used horseradish peroxidase-conjugated donkey anti-mouse (#715-035-150) and donkey anti-rabbit (#711-035-152) secondary antibodies from Jackson ImmunoResearch (West Grove, PA) at 1:20,000 dilution. IB with near-infrared fluorescent detection used primary antibodies from Cell Signaling Technology against phospho-ERK1/2 (#9101; 1:2000) and total-ERK1/2 (#9102; 1:2000). Primary antibodies against NKCC1 were rabbit T-NKCC (1:2000, #13884-1-AP; Proteintech) and sheep polyclonal antibodies against P-NKCC1: phospho Thr 203, Thr 207, Thr 212 (IB: 1:1000, #S763B) and phospho Thr 212, Thr 217 (IB: 1:1000, #S603D), both from the MRC Protein Phosphorylation and Ubiquitylation Unit, University of Dundee, (United Kingdom). Secondary antibodies (#925-68071, anti-rabbit and #925-32214, anti-mouse, both 1:15,000) were from Li-Cor Biosciences (Lincoln, NE). IF signals from transfected RH30 cells were visualized with goat-anti-mouse Alexafluor 350 (#A 21049) and goat anti-rabbit Alexafluor 568 (#A11036) conjugated antibodies, and the actin cytoskeleton was stained with Alexafluor 350- or 488-conjugated phalloidin (all from ThermoFisher Scientific, diluted 1:500). Nuclei were stained using 4,6-diamidine-2-phenylindole dihydrochloride (DAPI, Sigma-Aldrich).

### Animal models

All animals used in this study were bred, housed, and treated in accordance with standards set by the Animal Care and Use Committees at the University of Florida and the University of Massachusetts Medical School. Mice with a null mutation of the *Sgcg* gene were generated, bred, and phenotyped, as described previously [16]. Mice lacking the long muscle-specific archvillin (SV2) splice-form encoded by the *Svil* gene were generated by Ingenious Targeting Laboratory (Ronkonkoma, NY). The biochemical and functional consequences of this mutation are under investigation and will be described in detail elsewhere. In brief, the guide RNAs 5′-TGTAGGGCGATCCAAAGAAGAAG-3′ and 5′-TCTTCAATGCTTACCTGGCTCGG-3′ and CRISPR/Cas9 were used to generate a targeting vector in which the 609-nt exon 3 in the *Svil* gene, called exon 2 in the initial cDNA cloning paper [27], was replaced by an ivNeo selection cassette flanked by 759 bp and 838 bp of genomic sequence [42]. The targeting construct was verified by DNA sequencing, linearized with *NotI*, and electroporated into C57BL/6 embryonic stem (ES) cells. Targeted C57BL/6 FLP ES cells were microinjected into Balb/c blastocysts, and the resulting chimeras with a high percentage of black coat color were mated to C57BL/6 (WT) mice to generate germline Neo-deleted mice, which were identified by PCR screening of tail DNA. Neo-deleted mice were back-crossed four times with C57BL/6NCrl mice (Charles River Laboratories, Wilmington, MA) and screened by PCR for the absence of the FLP transgene and for proper integration at the *Svil* locus. Immunoblotting with rabbit polyclonal antibodies against archvillin N-terminal sequences confirmed the absence of full-length archvillin in skeletal muscle (Additional file 2, Fig. S1A).

### Mouse muscle plasma membranes

**Fig. 1:**
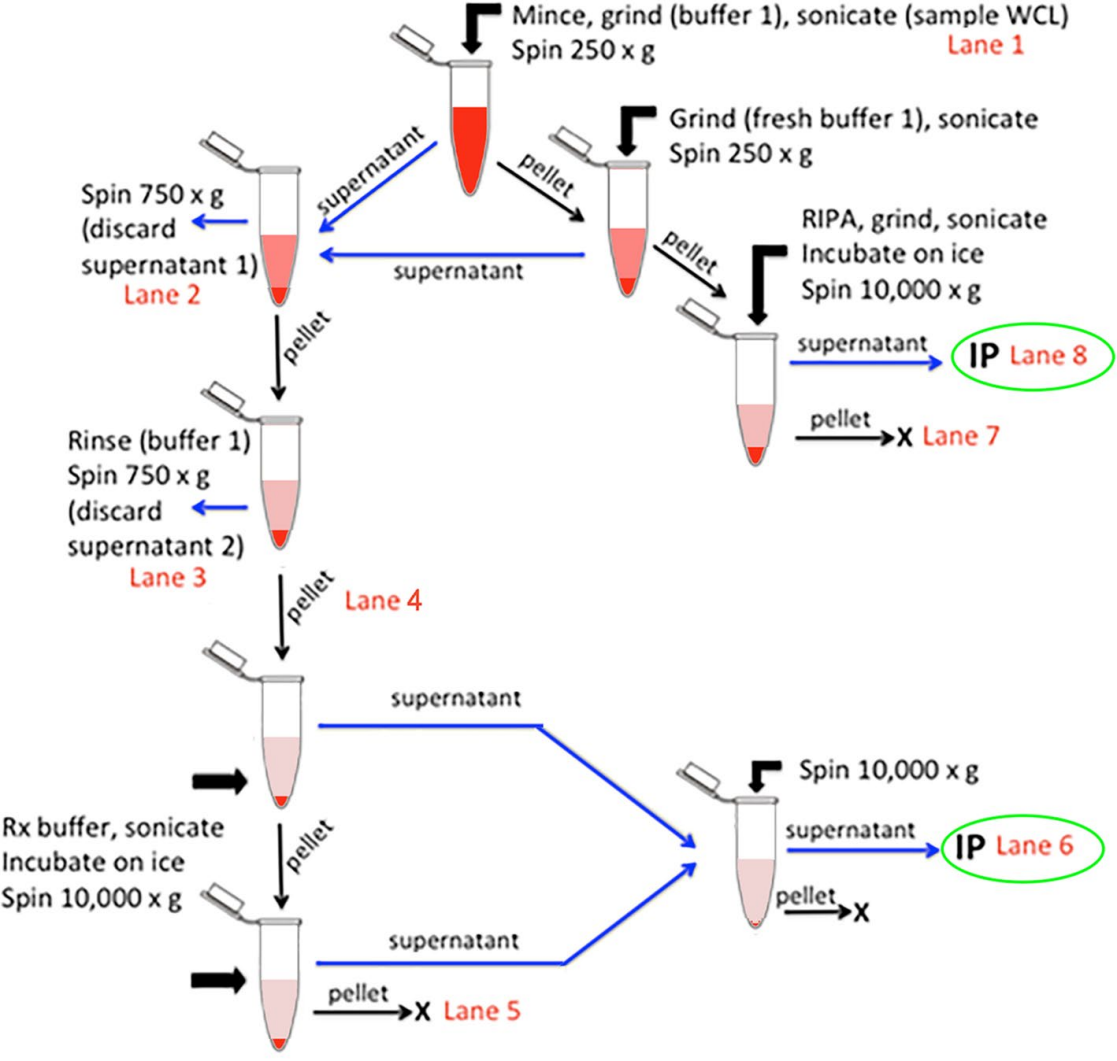
Flow chart for rapid membrane enrichment from mouse muscle. Skeletal muscle was fractionated as shown and described in detail in “Methods.” Lane numbers refer to analyses for total protein and immunoblotted sarcolemmal proteins, as shown in Fig. 2. Immunoprecipitations (IP, green ovals) with anti-Sgcg antibody were performed on extracts in either Rx buffer (high salt, 0.5% IGEPAL CA-630 in Rx buffer; lane 6) or with RIPA buffer (1% IGEPAL CA-630, 0.5% deoxycholate, 0.1% SDS; lane 8). WCL, whole cell lysate

Mouse gastrocnemius muscles were dissected and trimmed of extraneous tissue, cut into two ~ 100 mg pieces (wet tissue mass), and flash frozen in liquid nitrogen. To prepare for co-IP, one piece of frozen gastrocnemius muscle was very finely minced with a razor blade at room temperature on weighing paper while thawing, transferred to a 1.5-ml plastic mortar tube (#749520-0090, Kimble-Kontes, DWK Life Sciences, Millville, NJ), and processed according to a novel method adapted from those used to detect glucose transporter 4 [43], and other muscle membrane proteins [44–49] (Fig. 1). Minced muscles were covered with 900 μl of Buffer 1 (50 mM Tris, pH 8.0, 0.5 mM DTT, 0.1% NP-40, 10% glycerol, and protease inhibitor cocktail (Sigma-Aldrich #P8340) and lysed by manual grinding for 5–10 s with a blue plastic Kimble-Kontes pestle. Up to 4 samples were processed at one time, and all were stored on ice between steps. When all samples in a group were ground, they were immediately sonicated for 30 pulses with a microtip probe on a Branson Sonifier Cell Disrupter (Emerson Electric, Danbury, CT) with settings of 25% duty cycle, output ~ 1.5. A 100-μl portion of each sample was taken for gel analysis (Muscle Lysate, WCL). The remaining volumes were centrifuged at 250×*g* for 1 min at 4 °C. The cloudy supernatant was removed to a fresh 1.5-ml polypropylene microcentrifuge tube, and 450 μl of Buffer 1 was added to the remaining pellet, which was again ground manually for 5–10 s and sonicated. After another 1 min centrifugation at 250×*g*, this also-cloudy supernatant was combined with the first and centrifuged together at 4 °C for 10 min at 750×*g*. The remaining 250×*g* pellet was homogenized in an equivalent volume of 2x RIPA1 buffer (100 mM MOPS, pH 7.5, 0.3 M NaCl, 2% IGEPAL CA-630, 1% deoxycholic acid, 0.2% SDS, plus protease inhibitors) and incubated on ice while the other fractions were further processed; this incubation period was ≥ 25 min before sonication (see below). The supernatant from the 750×*g* spin was removed (Discard supernatant 1), and the remaining (white and somewhat fluffy) membrane pellet was resuspended by vortexing in 450 μl of Buffer 1. The membranes were again sedimented at 750×*g* for 10 min. The supernatant was removed (Discard supernatant 2), and the pelleted membranes were resuspended by briefly vortexing in 600 μl of cold Rx Buffer (25 mM MOPS, pH 7.5, 0.1 M NaCl, 0.5 M KCL, 10 mM MgCl_2_, 5 mM ATP (freshly made), 60 mM octylglucopyranoside, 0.5% IGEPAL CA-630, 10% glycerol, and protease inhibitors). The membranes were then sonicated three times, as above, with an incubation on ice of 1 min in between, until the suspension was slightly pearly. The pellets from the RIPA1-solubilized membranes also were sonicated once at this time, and both fractions were incubated on ice for 10 min after the last sonication. All tubes were centrifuged for 10 min at 10,000×*g*, and the supernatants removed to fresh tubes. The Rx membrane pellet was resuspended by briefly vortexing in 400 μl of Rx buffer and sonicated once before centrifugation at 10,000×*g* for 10 min. This final Rx pellet was resuspended in an equal volume of 2x Laemmli Sample Buffer [37]. The two Rx supernatants were combined and subjected to a final centrifugation at 10,000×*g* for 10 min. The RIPA1 pellet was resuspended in a Laemmli sample buffer volume equivalent to that of the corresponding RIPA supernatant.

### Light and confocal microscopy

Phase images of extracted muscle membranes were obtained with a Leica DMI 6000B inverted fluorescence microscope with a Leica DFC 365 FX camera, a Leica HCX PL Fluotar 10x/0.30 PH1 lens, and Leica Application Suite 3.2.0.9625 software (Leica Microsystems, Exton, PA). Immunofluorescent images were taken on the same system using a Leica HC PL APO 63x/1.40-0.60 oil lens. Images of fluorescently stained muscle sections were obtained on a Leica SP5 (II) AOBS laser scanning confocal microscope with a HCX PL APO CS 40.0×/1.30 oil UV lens and using Leica Application Suite Advanced Fluorescence (LAS-AF) 2.7.3.9723 software (Leica Microsystems CMS GmbH, Mannheim, Germany). Optical *z*-sections of 0.29 μm were obtained sequentially for each color channel through the muscle sample. Selected sequential sections comprising no more than 1.2 μm of *z*-thickness were processed using the Maximum Intensity Projection function in the software. All images were exported as TIF files, then uniformly adjusted and assembled with Adobe Photoshop CS3 software (Adobe Systems, Inc., San Jose, CA).

### Immunoblotting and analyses

Proteins were electrotransferred overnight onto Amersham™ Protran™ 0.45-μm nitrocellulose membranes (GE Healthcare Life Science, Marlborough, MA; #10600002). Chemiluminescent signals were visualized by SuperSignal West Pico or Femto reagents (ThermoFisher Scientific) on a Chemidoc MP Imaging System, using ImageLab™ software, version 4.1 (Bio-Rad Life Science Research, Hercules, CA). Densitometry of protein bands was determined using GelQuant.Net (version 1.7.8, BiochemLabSolutions.com; University of California, San Francisco, CA) software. Ratios of Unbound to Input signals were calculated using Microsoft Excel (Microsoft, Redmond, WA) and analyzed using GraphPad Prism 8.4.0 software (GraphPad Software, L.L.C., La Jolla, CA). Comparisons of multiple datasets were carried out using standard or non-parametric one-way ANOVAs, as indicated in the figure legends. Immunoblots were uniformly adjusted and assembled with Adobe Photoshop CS6 software.

For P/T-ERK1/2 and P/T-NKCC1 immunoblotting, *extensor digitorum longus* (EDL) muscles and *tibialis anterior* (TA) muscles were powdered using a mortar and pestle in dry ice and homogenized in RIPA2 buffer supplemented with PMSF and inhibitors of proteases (#P8340; Sigma-Aldrich) and phosphatases (#P5726; Sigma-Aldrich). Homogenates were incubated on ice for 60 min and centrifuged at 15,000×*g* for 15 min. Protein quantifications of muscle lysates were determined by the Bradford assay (#1863028; Thermo Fisher Scientific). For P/T-ERK1/2 analyses, EDL lysates (20 μg protein) were loaded in 4–12% Bis-Tris Midi Protein Gels (#WG1402A, Thermo Fisher Scientific) and electrotransferred to nitrocellulose. Each membrane was blocked for 90 min at room temperature with 5% BSA (BP9703100, Thermo Fisher Scientific) in TBS. Blots were incubated with anti-phospho-ERK1/2 and anti-total-ERK1/2 primary antibodies overnight at 4 °C, washed and incubated for 90 min at room temperature with secondary antibodies (Li-Cor Biosciences).

For P/T-NKCC1 analysis, TA lysates (20 μg protein) were loaded in 3–8% Bis-Tris Gel 1.0 mm (WG1002BOX; Thermo Fisher Scientific) and transferred to Immobilon-FL PVDF, 0.45-μm porosity (IPFL00010, EMD-Millipore) for 16 h at 40 mA in 10% methanol, 0.01% SDS, 0.1% NuPage Antioxidant (NP0005, Thermo Fisher Scientific). Each membrane was blocked for 120 min at room temperature with 5% BSA in TBS. Both P-NKCC antibodies were pre-incubated for 30 min with 10 μg/ml non-phospho peptide. After washing, blots were incubated for 90 min at room temperature with secondary antibodies (#925-68071, anti-rabbit; #925-32214, anti-goat/sheep; both at 1:15,000) from Li-Cor Biosciences. After washing, all blots were scanned with the Odyssey CLx Imaging System (Li-Cor Biosciences). The band intensity was automatically determined by the accompanying software Image Studio v.5.2 (Li-Cor Biosciences).

### Co-IPs from muscle membranes

For each IP from muscle lysates, 15 μg of antibody was used per 50 μl of Dynabeads Protein A (ThermoFisher Scientific; #10001D). Protein A-bound IgG was cross-linked using 20 mM dimethylpimelimidate (DMP, Pierce/ThermoFisher Scientific), as previously described [35]. The Rx membrane lysate was diluted with an equal volume of Rx buffer without KCL or ATP (25 mM MOPS, pH 7.5, 0.1 M NaCl, 10 mM MgCl_2_, 60 mM octylglucopyranoside, 0.5% IGEPAL CA-630, 10% glycerol), and the RIPA1 extract was diluted to 750 μl using RIPA1 buffer. Both the diluted Rx and RIPA1 lysates were pre-cleared with 20 μl of Dynabeads Protein A (pre-rinsed in Rx or RIPA1 buffer) for 30 min at 4 °C. A 100-μl aliquot was removed as “Input” before proceeding with each IP. The lysates were divided evenly among the IP antibodies (200 μl each for RIPA1, 300 μl each for Rx), and incubated at 4 °C for 2 h with gentle rotation. Dynabeads were collected magnetically, and the Unbound supernatants were transferred to a fresh tube. The beads were rinsed 5 times with 500 μl of ice-cold 0.5 × TBST (5 mM Tris, pH 7.5, 83 mM NaCl, 0.05% Tween-20); beads were transferred to a fresh tube at the second rinse. Immunoprecipitated proteins were eluted under non-reducing conditions in Laemmli sample buffer lacking dithiothreitol (DTT) [37], and the eluate was transferred to a fresh tube.

### In-gel protein digestion and LC-MS/MS analysis

IPs and analyses were performed using triplicate biological samples for each antibody and muscle genotype. *Gastrocnemius* muscles containing the SC were from C57BL/6 (WT) mice and a new mouse strain homozygous for the genetic ablation of a large 5′ coding exon in the *Svil* gene (*Svil*^*−/−*^). Negative controls were *gastrocnemius* muscles from *Sgcg*^*−/−*^ mice [16] and IPs with nonspecific rabbit IgG. DTT (final concentration of 2 mM) was added to the immunoprecipitated samples before they were electrophoresed into a 10% Laemmli resolving gel [37], without a stacking gel, until the dye front was ~1 cm below the bottom of the loading wells; at least two blank wells separated samples. Gel proteins were then visualized with the Invitrogen Novex Colloidal Blue Staining Kit, according to the manufacturer's protocol (ThermoFisher Scientific; #LC6025). Each IP sample was excised as a single gel piece. Gel pieces were subjected to in-gel trypsin digestion after reduction with DTT and alkylation with iodoacetamide. Peptides eluted from the gel were lyophilized and re-suspended in 25 μl of 5% acetonitrile with 0.1% (v/v) trifluoroacetic acid (TFA). Using an injection volume of 3 μl, peptides were loaded by a Waters nanoAcquity UPLC (Waters Corp., Milford, MA) in 5% acetonitrile (0.1% formic acid (v/v)) at 4.0 μl/min for 4.0 min to a 100 μm I.D. fused-silica pre-column (Kasil frit) packed with 2 cm of 5 μm (200Å) Magic C18AQ (Bruker-Michrom, Billerica, MA). Peptides were then eluted at 300 nl/min via a 75-μm I.D. gravity-pulled analytical column packed with 25 cm of 3 μm (100Å) Magic C18AQ using a linear gradient from 5-35%B (mobile phase A, water + 0.1% (v/v) formic acid; mobile phase B, acetonitrile + 0.1% (v/v) formic acid) over 90 min. Ions were introduced by positive electrospray ionization via liquid junction electrode into a Thermo Scientific Q Exactive hybrid mass spectrometer. Mass spectra were acquired over *m/z* 300-1750 at 70,000 resolution (*m/z* 200) using an AGC (automatic gain control) target ion population of 1e6. Data-dependent acquisition selected the top 10 most abundant precursor ions for tandem mass spectrometry using higher-energy C-trap dissociation (HCD) using an isolation width of 1.6 Da, a normalized collision energy of 27, a maximum ion fill time of 110 ms, and an AGC target ion population of 1e5. Tandem mass spectra were acquired at 17,500 (*m/z* 200) resolution.

### Proteomics data analysis

Raw data files were peak processed with Proteome Discoverer (version 1.4, Thermo Scientific) followed by identification using Mascot (version 2.5, Matrix Science, Boston, MA) against the SwissProt *Mus musculus* database (download 04/2018). Search parameters included full trypsin specificity with up to 2 missed cleavages, fixed modification of cysteine carbamidomethylation, and variable modifications of methionine oxidation, glutamine to pyroglutamic acid conversion, and protein N-terminal acetylation. Assignments were made using a 10 ppm mass tolerance for the precursor and 0.05 Da mass tolerance for the fragments. All non-filtered search results were processed by Scaffold software (version 4.4.4, Proteome Software, Inc., Portland. OR), utilizing the Trans-Proteomic Pipeline (Institute for Systems Biology, Seattle, WA). Rx extracts and RIPA1 extracts were analyzed separately. For both extracts, threshold values were set at 80% for peptides (false-discovery rates (FDR) of 0.45% for Rx extract and 0.53% for RIPA extracts) and 99% for proteins (2 peptide minimum; Rx extracts, FDR 20.0%; RIPA extracts, FDR 12.0%). Quantitative comparisons were made in the Scaffold software, using normalized weighted spectra, with an ANOVA significance level *P* < 0.05 without a post-test. Application of a Benjamini-Hochberg multiple test correction eliminated as significant all of the Rx buffer interactors and β-sarcoglycan and all proteins with higher *P* values in RIPA1 buffer, but the overall *P* values were unchanged. Interactors were listed in order of increasing *P* value from both Rx (Additional file 3, Table S2) and RIPA (Additional file 4, Table S3) extracts and edited manually for candidate Sgcg interactors, as described below.

Proteins specifically co-immunoprecipitating with Sgcg were defined as those that had significantly higher total normalized weighted spectral counts in IPs from C57BL/6 wild-type muscles and/or from Svil-targeted (*Svil*^*−/−*^) muscles, as compared to spectral counts from *Sgcg*^*−/−*^ muscles and spectral counts in IPs with control IgG from any muscle type. Spectral counts were normalized based on each protein’s predicted molecular mass. “Top candidate interactors” were represented by ≥ 3 total normalized spectral counts from WT or *Svil*^*−/−*^ muscles and were selected based on *P* values < 0.05 and quantitative profiles that showed increases of total normalized spectral counts that were ≥ 2-fold over those in each of the two types of negative controls. In most cases, total normalized spectral counts were greatly reduced in *Sgcg*^*−/−*^ muscles and very few counts were observed with the IgG control antibody. “Other candidate interactors” also had *P* values < 0.05, but were below the 2-fold spectral count threshold or were elevated only in *Svil*^*−/−*^ muscles. Two proteins (myotilin, titin) made this cut-off when the high background counts observed with control IgG were subtracted first. Because titin and myotilin mutations are responsible for LGMD, type 2J and type 1A, respectively [50], we drew the cut-off for statistical significance at *P* < 0.05 under these conditions. Exceptions were made for α- and β-sarcoglycans, known to be part of the SC [51], and for candidate PP1β-binding interactors close to the arbitrary 2-fold cut-off. A group of F-actin-binding proteins, e.g., spectrin, dystrophin, and filamin C, were recovered at approximately equal abundance from all three muscle types with anti-Sgcg, but not with control IgG, and were not considered further (Additional files 3 and 4; Tables S2 and S3).

This experimental approach may be inherently limited by binding of residual actin filaments to IgG [52], leading to high backgrounds of F-actin-binding proteins. Co-IPs also were performed with affinity-purified antibodies against the actin-binding protein archvillin (anti-mAV) and Rx and RIPA1 extracts of WT muscle and muscles deficient in archvillin. No archvillin-associated candidate interactors were identified because of high background binding with rabbit IgG.

Direct and indirect structural and signaling relationships among the candidate Sgcg interactors were identified using the Connect command in Ingenuity Pathway Analysis (IPA) Path Designer software, version 01-14 (Qiagen Bioinformatics, Redwood City, CA) Sgcg interactions with Svil [26] and NKCC1 were added manually and positioned with the IPA Auto-Layout command. The B-Raf, MEK1/2, and ERK1/2 signaling cascade was added manually, along with their connections to sarcolemmal proteins. Interactor colors and designations were manipulated for clarity using Adobe Photoshop CS6 (Adobe Systems, Inc.).

### Plasmids

Elizabeth McNally (Northwestern University Feinberg School of Medicine, Chicago, IL) generously provided the plasmid encoding untagged murine Sgcg [36]. Myc-DDK (Flag)-tagged murine β- (#MR204617), δ- (#MR221060) and γ- (#MR223013) sarcoglycan cDNAs were from OriGene Technologies (Rockville, MD), as were Myc-DDK-tagged cDNAs encoding mouse Ppp1r12b/MYPT2 (#MR226968) and mouse tensin 2 (tensin like C1 domain-containing phosphatase, isoform 1/Tenc1; #MR211954). The plasmid encoding EGFP-PP1β/δ (EGFP-C1 vector) was obtained from Mathieu Bollen, (KU Leuven, Leuven, Belgium), via A. J. Baucum (Indiana University-Purdue University, Indianapolis, IN) [53]. Plasmid encoding human PP1β-EGFP (EGFP-N3 vector) was a gift from Angus Lamond and Laura Trinkle-Mulcahy (Addgene plasmid #44223; http://n2t.net/addgene:44223; RRID:Addgene_44223) [54]. EGFP-tensin2 (human) was a gift from David Critchley and Kenneth Yamada (Addgene plasmid #105298; http://n2t.net/addgene:105298; RRID:Addgene_105298) [55]. HA-tagged human NKCC1 (pdDNA3.1-HA-CFP-hNKCC1 WT (NT15-H)) was a gift from Biff Forbush (Addgene plasmid #49077; http://n2t.net/addgene:49077; RRID:Addgene_49077 [56]).

GST-bSV1398-1792 was described previously [57]. A pIRES2-EGFP vector containing full-length human Sgcg cloned between the EcoRI and BamHI sites [58] was used as a template to create plasmids encoding only the 35 N-terminal residues fused with C-terminal EGFP. The vector was first modified to contain an internal BamHI site by converting Leu-36 to glycine and Tyr-37 to serine using the QuikChange Site-Directed Mutagenesis kit (Agilent Technologies; #200519) and the L36Y37-BamHI-sense and L36Y37-BamHI-antisense primers in Additional file 1, Table S1. The modified vector was restriction digested with BamHI and NotI, and the similarly digested EGFP cassette from pEGFP-N3 was directionally ligated to be in-frame with the Sgcg cytoplasmic domain, i.e., residues 1–35. The Sgcg1-35-EGFP vector was then used as a template to generate hSgcg-1-35-EGFP fragments to clone into either the pET30a vector for His-tagging using the Sgcg-BglII-start-For and Sgcg-EcoRI-end-Rev primer pair or into the pMALc5x vector (New England BioLabs, Beverly, MA; #N8108S) for maltose-binding protein tagging using the Sgcg-For-EcoRV-1Met and Scgc-EcoRI-end-Rev primer pair. EGFP only controls were cloned into the same vectors using a BamHI-NotI double digest from pEGFPN3 into pET30a, and an XmnI-EcoRI double digest from pET30a-EGFP into pMALc5x. Vectors containing wild-type hSgcg-1-35-EGFP were then mutated at Tyr-6 to Ala using the QuikChange mutagenesis kit and the Scgc-Y6A-sense and antisense primer pair. All restriction enzymes were from New England BioLabs (Beverly, MA).

The plasmid encoding 3X-HA-tagged human NKCC1 synthetic cDNA with convenient restriction sites [56] was modified in two ways. First, we eliminated the sequence encoding all of the transmembrane domains and much of the cytoplasmic domains with a double restriction digest using *HpaI* and *EcoRV*. We then re-ligated the blunt ends to generate 3xHA-CFP-hNKCC1-cyto, which encodes the 3xHA-CFP tag, followed by the cytosolic NKCC1 N-terminal amino acids Glu-2 through Val-141 and the 91 residues of the cytosolic NKCC1 C-terminus (I-1118 through S-1196) [56]. Second, we generated a plasmid encoding a control 3XHA-CFP protein, which lacks the entire NKCC1 sequence, by inserting a TAA stop codon immediately downstream of the CFP-coding sequence using PCR with the QuikChange® II XL site-directed mutagenesis kit (Agilent Technologies; #200521) and primers HA-CFP-Stp For and HA-CFP-Stp Rev (Additional file 1, Table S1). All plasmids were verified by end sequencing.

### Cell culture, screens with exogenously expressed proteins, and proximity ligation assay

Human RH30 rhabdomyosarcoma cells (SJC-RH30, American Type Culture Collection #CRL-2061, Manassas, VA) were maintained at 37 °C and 5% CO_2_ in RPMI-1640 medium modified to contain 2 mM L-glutamine, 10 mM HEPES, 1 mM sodium pyruvate, 4500 mg/L glucose, 1.5 g/L sodium bicarbonate (#A1049101, Thermo Fisher Scientific), supplemented with 10% heat-inactivated fetal bovine serum, an additional 2 mM L-glutamine, and Pen-Strep. Transfections were done in 6-cm dishes that had been plated the day before so that the cells would be ≤ 60% confluent, using Lipofectamine 2000 (Thermo Fisher Scientific) and a scaled-down version of the manufacturer’s instructions. First, an equimolar mixture of C-terminally myc-DDK-tagged β-- δ-- and γ-sarcoglycan cDNAs was made in water at a total concentration of 0.3 μg/μl. For co-transfections in a 6-cm dish, with or without coverslips, 5 μl of this mix (1.5 μg) or 1.5 μg of an empty Flag vector control were then mixed with 1.5 μg of the other mammalian expression plasmid before dilution to 250 μl in OptiMEM reduced-serum medium (#31985, Thermo Fisher Scientific). Concurrently, 10 μl of Lipofectamine 2000 per transfection were diluted to 250 μl in OptiMEM. Both solutions were incubated for 5 min at room temperature and then combined (500 μl per transfection dish) and incubated at room temperature for an additional 10 min. The culture medium in each plate was replaced with 4.5 ml of fresh medium immediately before the plasmid DNA complexes in OptiMEM were added drop-wise across each plate. Plates were then incubated for 3 to 4 h at 37 °C and 5% CO_2_ before the medium was replaced with 5 ml of fresh growth medium and the incubation was continued overnight. Cells on coverslips in 6-well plates were cultured and transfected the same way, except using half the amounts of plasmid DNA and Lipofectamine 2000 in 100 μl of OptiMEM and a total volume of 2 ml medium per well. Cells were harvested 23 to 25 h post-transfection for either co-IP or fixation for immunofluorescence or proximity ligation assay (PLA). Fixation was performed as previously described [59], using either ice-cold methanol for 15 min or 4% paraformaldehyde in CSK buffer (10 mM PIPES, pH 6.8, 300 mM sucrose, 100 mM sodium chloride, 3 mM magnesium chloride, 1 mM EGTA [60]) for 30 min on ice. Pre-extraction experiments involved treating cells on coverslips with 0.1% Triton X-100 in CSK buffer for 4 min on ice and rinsing briefly with PBS before fixation and immunofluorescence imaging, as described above.

PLA was performed using the Duolink In Situ Orange kit (Sigma #DUO92102), according to the manufacturer’s instructions, with mouse anti-HA (Cell Signaling #2367, 1:200) and rabbit anti-Sgcg (Proteintech #18102-AP, 1:200) as primary antibodies. PLA speckles were counted by hand after signal inversion and ~3-fold enlargement using Adobe Photoshop. Nuclei associated with zero speckles were assumed to be untransfected cells. Graphical and statistical analyses were performed with GraphPad Prism.

### Co-IPs and supernatant-depletion assays

Standard co-IP experiments and supernatant-depletion assays were carried out after extraction of RH30 cells with modified RIPA1 (no SDS). Cells between passage 5 and 28 in 6-cm dishes were transfected for these experiments as follows: (1) with a mixture of Myc-DDK (Flag)-tagged murine β-, δ-, γ-sarcoglycans and either HA-CFP, HA-CFP-Cyto, or HA-CFP-NKCC1; or (2) with Myc-Flag-tagged sarcoglycans, or an empty Flag vector, and either PP1β-EGFP, EGFP- PP1β or EGFP. Transfected cells were grown for 24 h and were 50–60% confluent when extracted on ice for 15 min in 350 μl of pre-chilled modified RIPA1 buffer (no SDS), plus protease and phosphatase inhibitors (# P8340, P2850, P5726, Sigma-Aldrich). Extracts were collected by scraping and transferred to 1.5-ml polypropylene centrifuge tubes, sonicated for 25 pulses with a Branson sonifier, as above, and centrifuged at 18,000×*g* for 10 min at 4 °C. The resulting supernatants were removed to a fresh 1.5-ml tube and used for either co-IPs or supernatant-depletion assays.

For experiments with HA-tagged proteins in RH30 cell lysates, Protein A Dynabeads were incubated for 1 h at room temperature either with monoclonal rabbit anti-HA antibody, clone C29F4, (for HA-CFP-NKCC1 experiments), or with rabbit polyclonal anti-Flag. Beads were rinsed once with RIPA1 buffer and split into equal fractions. The rinse was removed from each tube, and the beads were resuspended in 300 μl of each cell extract and incubated with rotation for 1 h at 4 °C. For experiments involving EGFP, 25 μl of GFP-Trap Dynabeads (product code gtd, Chromotek Inc., Islandia, NY, USA) were used per IP and washed once with ice-cold RIPA1 buffer before the addition of cell extract.

Co-IPs and supernatant-depletion assays were carried out similarly, except co-IPs focused on the amounts of proteins in pellets (“Bound”) and supernatant-depletion assays focused on the differences in protein concentration before (“Input”) and after (“Unbound”) centrifugation. The latter approach better detects low-avidity interactions [61]. In supernatant-depletion assays, either 20 μl of beads with bound anti-HA antibody or 25 μl of GFP-Trap Dynabeads were mixed with 300 μl of each cell extract for 1 h at 4 °C. A 100-μl “Input” sample was taken, and the remainder of each mixture was quickly sedimented through a cushion of 20% sucrose in RIPA1 buffer using narrow-bore BioRad tubes (#223-9502) and a swinging-bucket rotor at 800×*g* for 5 min. The top 100 μl was taken as the “Unbound” sample. Input and Unbound samples were resolved on 12% SDS-polyacrylamide gels and electroblotted.

Supernatant-depletion assays [61] also were carried out with purified, recombinant N-terminally tagged archvillin C-terminus and C-terminally tagged Sgcg cytoplasmic domain. The GST-bSV1398-1792 protein is respectively 94.43% and 96.96% identical to the corresponding sequences in murine and human archvillins (from Clustal2.1, Conway Institute UCD, Dublin, Ireland), and was made as described previously [57]. All recombinant EGFP proteins were made in Rosetta 2 pLyS bacteria (#71400, Novagen/Sigma-Aldrich), as described for antigen production. His-EGFP-tagged proteins were purified via NiNTA agarose (Qiagen), and MBP-tagged proteins were purified via amylose (New England BioLabs), according to the manufacturers’ instructions. We removed the tags from both sets of proteins (thrombin digestion for His tags, Factor Xa proteolysis for MBP), and re-purified them with NiNTA agarose or by ion-exchange chromatography, respectively. GST-bSV-1398-1792 was mixed with the EGFP proteins in a total volume of 250 μl and incubated for 30 min at 4 °C with rotation before the addition of 25 μl of glutathione-Sepharose beads (#17-0756-01, GE Healthcare). After an additional 30-min incubation, 100 μl of the total slurry was removed as the “Input” sample; the rest was loaded atop a 25% sucrose cushion in a narrow diameter tube and centrifuged, as described above. Input and Unbound samples were resolved on 12% SDS-polyacrylamide gels and electroblotted.

### Muscle immunostaining

To determine the localization of NKCC1 in EDL muscles, 10-μm cryosections were obtained from EDL muscles of C57 and *Sgcg*^*−/−*^ mice. Both longitudinal and cross sections were utilized. Sections were washed thrice in PBS, 10 min per wash. Sections were fixed for 5 min in 4% paraformaldehyde in PBS, followed by permeabilization with 0.5% Triton-X, PBS. Following blocking in 5% bovine serum albumin (BSA) in PBS for 1 h at room temperature, sections were incubated overnight at 4 °C with primary antibodies diluted in 5% BSA against dystrophin (MANDYS1, 3B7) [39] and NKCC1 (1:500 NKCC1, rabbit pAb 13884-1-AP; Proteintech) or with 5% BSA alone as a negative control. Sections were then washed thrice in PBS, 10 min/wash, and incubated 1 h at room temperature in the dark with secondary antibodies diluted in 5% BSA (1:10,000, Alexafluor 488 IgG anti-mouse (#A11029); 1:1000 Alexafluor 568 IgG anti-rabbit (#A11036) (Invitrogen). Sections were washed again in the dark (PBS 3 times, 10 min each), air-dried, and covered with a mounting agent (ProLongTM Diamond Antifade with Dapi, Cat#P36962, Thermo Fisher Scientific) and coverslip.

### NKCC1 inhibition in vivo

EDL muscles from C57 or *Sgcg*^*−/−*^ mice (9-14 wks old) were isolated and prepared for muscle mechanics as previously described [62]. EDLs with suture loops were mounted to the 800A in vitro Muscle Apparatus (Aurora Scientific, Ontario, CAN) via a rigid hook and hook to a servomotor arm. The muscles were positioned between two platinum plate electrodes, and in a Radnoti vessel filled with Ringer’s solution equilibrated with 95% O_2_/5% CO_2_ and maintained at 22 °C. EDL stimulation was controlled and carried out using Aurora Scientific (Ontario, CAN) hardware and software (i.e., High Power Bi-Phase Current Stimulator, 300C Dual-Motor Level System, Digital Controller Interface, and DMC v5.420). Following determination of optimal length (Lo), muscles were subjected to three bouts of supramaximal stimulation at 150 Hz for 500 ms, separated by 3 min to obtain maximum isometric force (Max Force). After completion of initial stimuli, EDLs were treated with bumetanide dissolved in 100% ethanol (final concentration 50 μM bumetanide, 1% ethanol) or ethanol vehicle alone (final concentration 1% ethanol) for 20–30 min. A second series of Max Force measurements were conducted to determine potential changes in twitch and tetanic forces after treatment. After a 5-min rest period, EDLs underwent five eccentric contractions, consisting of a 10% Lo lengthening during the final 200 ms of stimulation, with a 5-min rest interval between stimuli. A 30-min wait period followed muscle mechanics. EDLs were then lightly dried, weighed, snap frozen, and stored at − 80 °C. For those EDLs remaining at rest, they were pinned loosely in a petri dish with oxygenated Ringers solution containing either 50 μM bumetanide, 1% ethanol, or neither substance for 30 min. Similarly, non-mechanics EDLs were snap frozen afterwards and stored at − 80 °C for future biochemical and histological analyses.

### P/T-ERK1/2 and P/T-NKCC1 activation assays

EDL and TA muscles were powdered using a mortar and pestle in dry ice and homogenized in RIPA2 buffer supplemented with PMSF and inhibitors of proteases (P8340; Sigma-Aldrich) and phosphatases (P5726; Sigma-Aldrich). Homogenates were incubated on ice for 60 min and centrifuged at 15,000×*g* for 15 min. Protein quantifications of muscle lysates were determined by the Bradford assay (#1863028; Thermo Fisher Scientific). For P/T-ERK1/2 analyses, EDL lysates (20 μg protein) were loaded in 4–12% Bis-Tris Midi Protein Gels (#WG1402A, Thermo Fisher Scientific) and electrotransferred to nitrocellulose. Each membrane was blocked for 90 min at room temperature with 5% BSA (BP9703100, Thermo Fisher Scientific) in TBS. Blots were incubated with anti-phospho-ERK1/2 and anti-total-ERK1/2 primary antibodies overnight at 4 °C, washed and incubated for 90 min at room temperature with secondary antibodies (Li-Cor Biosciences).

For P/T-NKCC1 assays, TA lysates (20 μg protein) were loaded in 3–8% Bis-Tris Gel 1.0 mm (WG1002BOX; Thermo Fisher Scientific) and transferred to Immobilon-FL PVDF, 0.45-μm porosity (IPFL00010, EMD-Millipore) for 16 h at 40 mA in 10% methanol, 0.01% SDS, 0.1% NuPage Antioxidant (NP0005, Thermo Fisher Scientific). Each membrane was blocked for 120 min at room temperature with 5% BSA in TBS. Both P-NKCC antibodies were pre-incubated for 30 min with 10 μg/ml non-phospho peptide. After washing, blots were incubated for 90 min at room temperature with secondary antibodies (#925-68071, anti-rabbit; #925-32214, anti-goat/sheep; both at 1:15,000) from Li-Cor Biosciences. After washing, all blots were scanned with the Odyssey CLx Imaging System (Li-Cor Biosciences). The band intensity was automatically determined by the accompanying software Image Studio v.5.2 (Li-Cor Biosciences).

## Results

### Identification of new candidate sarcoglycan interactors

**Fig. 2:**
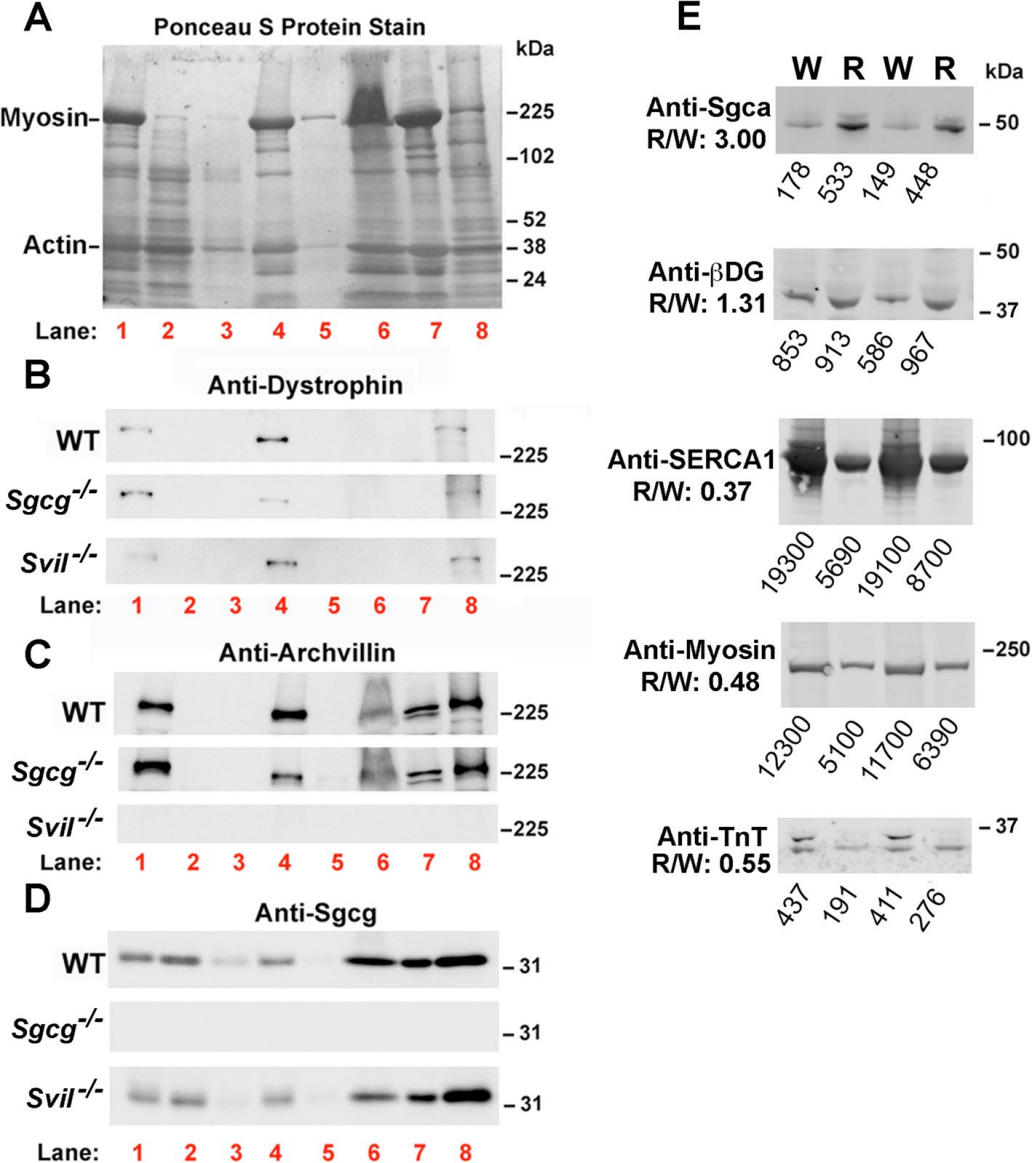
Sarcolemmal proteins recovered from Rx (lane 6) and RIPA1 (lane 8) buffers. Gastrocnemius muscles (~ 100 mg) from either C57BL/6 (WT) mice or from mice lacking γ-sarcoglycan (Sgcg^−/−^) or full-length archvillin (Svil^−/−^) were fractionated as shown in Fig. 1; lane numbers in red correspond with Fig. 1. Blots were stained for **A** protein with Ponceau S and probed for the sarcolemmal proteins **B** dystrophin, **C** archvillin, and **D** γ-sarcoglycan (Sgcg, using our new antibody). Much actin is removed by the low-salt in buffer 1 (**A**, lanes 2–3); most myosin II is extracted by the high salt in Rx buffer (**A**, lane 6) or remains insoluble in RIPA1 buffer (**A**, lane 7). Blots are representative of 3 biological replicates per genotype. Fractions 1–6 are normalized to total protein; fractions 7–8 are normalized by volume to each other; total protein in fraction 8 approximates that of fraction 1. Lane 1, whole muscle extracts. Lanes 2–3, discarded washes. Lane 4, enriched membranes before extraction. Lane 5, discarded Rx pellet. Lane 6, Rx supernatants. Lane 7, discarded, insoluble RIPA1 pellets. Lane 8, RIPA1 supernatants. **E** Comparison of protein abundance by immunoblotting of whole tissue lysates (W) vs. RIPA1 lysates (R). Equal protein loading (based on BCA quantification) for each condition was used. DGC proteins Sgca and β-dystroglycan (β-DG) were enriched in RIPA1 extracts of wild-type C57BL/6 muscles (equivalent to lane 8 in **A**–**D**), as compared with whole muscle lysates (equivalent to lane 1 in **A**–**D**), whereas the sarcoplasmic reticulum marker SERCA1 and the contractile apparatus proteins myosin II and fast troponin T (TnT) were depleted. Mean band intensities from ImageStudio software are indicated below each lane and used to calculate the ratios of RIPA1 to whole muscle lysate (R/W) shown to the left of each blot

To complement previous two-hybrid screens for the identification of sarcoglycan interactors [25, 26], we used a new co-immunoprecipitation (co-IP) and label-free proteomic approach to identify proteins directly or indirectly associated with Sgcg (Fig. 1). Eliminating time-consuming ultracentrifugation and carbohydrate-crosslinking lectins [46, 47], we modified the method of Yamamoto et al. [43] to enrich sarcolemmal proteins from a single murine muscle (50 mg to 100 mg wet mass), using bench-top equipment available in most research laboratories. Our method avoids potentially false associations due to carbohydrate agglutination by an extracellular lectin, but sacrifices the high sarcolemmal purity of previous approaches. Our relatively rapid approach takes less than 3 h for up to 4 samples from individual mouse muscles (Fig. 1). Sgcg-associated proteins were interrogated after extraction of relatively low-density membranes with a cytoskeletal disruption and relaxation (Rx) buffer and after extraction of higher-density, cytoskeleton-associated fractions with an SDS-containing RIPA1 buffer (Figs. 1 and 2). To our knowledge, the higher-density, cytoskeleton-associated fractions have not been explored for sarcoglycan- or other DGC-associated proteins. Each detergent supernatant contained the sarcolemmal marker proteins dystrophin, archvillin, and Sgcg (Fig. 2B–D, lanes 6, 8). The RIPA1 extract was enriched in proteins associated with the DGC (dystrophin, archvillin, Sgcg, Sgca, β-dystroglycan) (Fig. 2B–E), and proteins associated with the sarcoplasmic reticulum (SERCA1) and contractile apparatus (actin, myosin II, troponin-T) were reduced (Fig. 2A, E). The Rx supernatants contained more and larger particulates than did the RIPA supernatants, but no large membrane sheets remained (Additional file 5, Fig. S2).

**Fig. 3:**
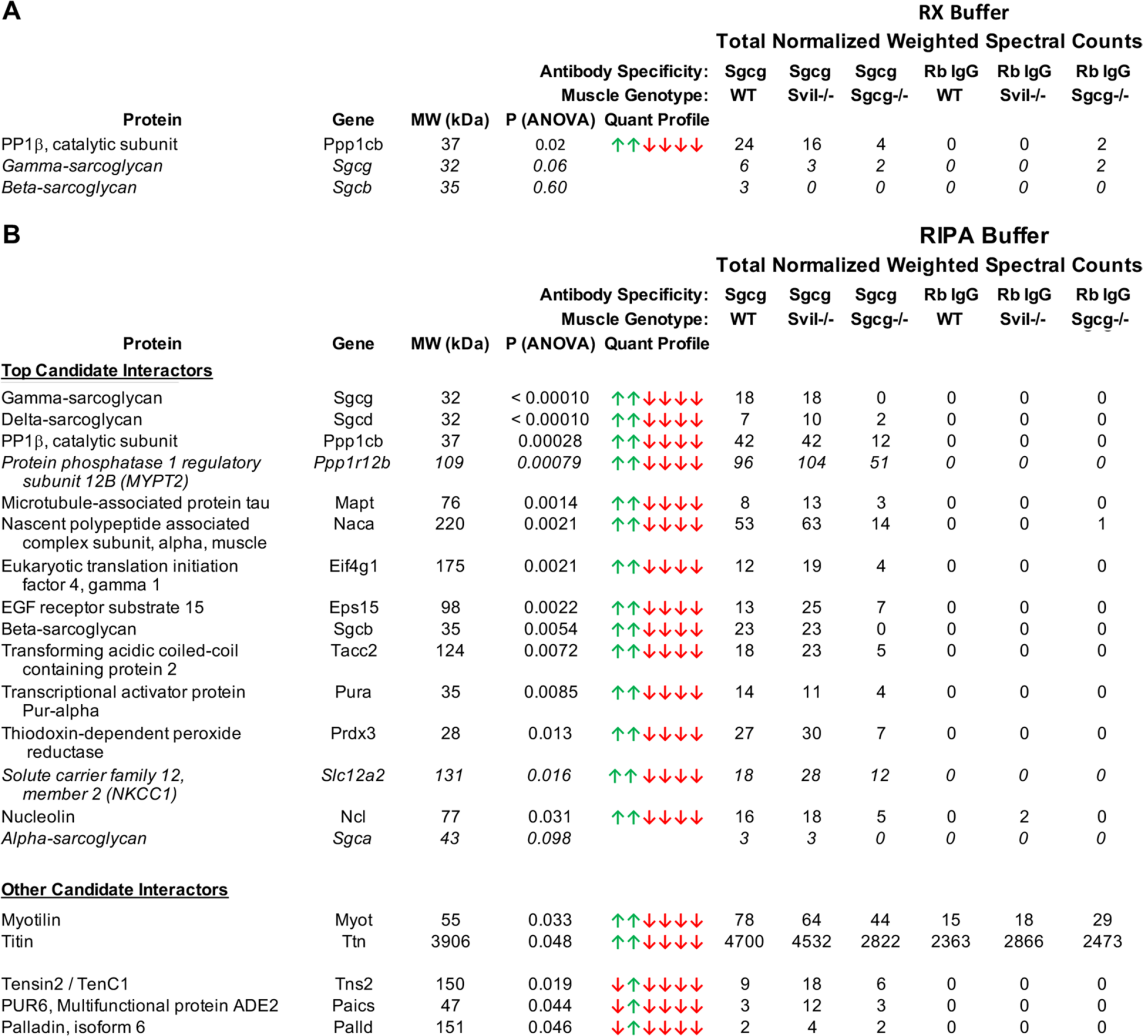
Proteins immunoprecipitating with Sgcg. Total normalized spectral counts were determined by adding counts from triplicate biological replicates of co-IPs from Rx or RIPA extracts, weighted for differences in protein mass. Increases in protein abundance were identified by ANOVA (green arrows), as compared with samples with statistically fewer spectral counts (red arrows); arrow order corresponds to the columns of total normalized weighted spectral counts. Candidate direct or indirect interactors, selected as described in “Methods,” were ranked in order of increasing *P* value. **A** Only protein phosphatase 1B, catalytic subunit (PP1β)) was significant in Rx buffer. **B** Top Candidate Interactors from RIPA1-solubilized membranes were defined as those recovered with anti-Sgcg antibody from both muscle genotypes containing Sgcg (WT, *Svil*^−*/*−^) and represented by fewer than half as many total normalized weighted spectral counts in each of the two sets of control IPs. One control was IPs with anti-Sgcg antibody from *Sgcg*^−*/*−^ muscles; the second was IPs with a nonspecific rabbit IgG (Rb IgG) from muscle of any genotype. Other Candidate Interactors with *P* values < 0.05 either exhibited higher than expected background counts or were significantly enriched only in *Svil*^−*/*−^ muscles. Values for sarcoglycans not flagged as statistically significant are shown in italics for comparison with the newly identified candidate Sgcg interactors. The candidate interactors MYPT2 and NKCC1 also are italicized because the numbers of counts in *Sgcg*^−*/*−^ muscle just miss the arbitrary 50% threshold. Other Candidate Interactors fell into two categories. Myotilin and titin met the *Sgcg*^−*/*−^ muscle cut-off criterion if the average of the backgrounds observed with control IgG were first subtracted from total normalized counts. Tensin 2, PUR6, and palladin met criteria only for *Svil*^−*/*−^ muscle

We identified new candidate direct or indirect interaction partners for the SC by comparing proteins recovered using our new high-avidity anti-Sgcg antibody crosslinked onto magnetic beads with those obtained from negative controls (Fig. 3; Additional files 3, and 4; Tables S2 and S3). *Gastrocnemius* muscles containing the SC were from C57BL/6 (WT) mice and a new mouse strain homozygous for the genetic ablation of a large 5′ coding exon in the *Svil* gene (*Svil*^*−/−*^). Negative controls were *gastrocnemius* muscles from *Sgcg*^*−/−*^ mice [16] and IPs with nonspecific rabbit IgG.

All four sarcoglycans were immunoprecipitated with anti-Sgcg from the WT and *Svil*^*−/−*^ RIPA1 extracts although the *P* value for Sgca, the least tightly incorporated sarcoglycan subunit [63, 64], was only 0.095, due to a low number of specific spectral counts (Fig. 3). These results are consistent with the previous observation that the SC is stable in SDS-containing buffers [65] and suggest that the other specifically co-immunoprecipitating proteins identified here (Fig. 3) are excellent new candidate interactors for the SC.

The top co-immunoprecipitating, non-sarcoglycan protein in both Rx and RIPA1 extracts is the Ser/Thr protein phosphatase catalytic subunit 1B (PP1β, also called PP1δ) (Fig. 3). Additional candidate interactors emerged from the RIPA1 extract of sarcolemmal proteins associated with large detergent-resistant structures (Fig. 3A vs. B). Because PP1β was the top candidate, the presence of MYPT2, NKCC1, and tensin2, which are known or predicted to bind to PP1β, also was intriguing (Fig. 3). The N-terminal tail of the Na^+^-K^+^-2Cl^−^ symporter (NKCC1, gene *Slc12a2*) can scaffold PP1β and a regulatory kinase for reciprocal control of NKCC co-transport activity [66]. NKCC1 supports the opening of voltage-activated Ca^++^ channels by accumulating Cl^−^ ions in the cytosol through cotransport with extracellular Na^+^ and K^+^ lost during active muscle contraction [67]. MYPT2, encoded by the *Ppp1r12b* gene, concentrates PP1 catalytic subunits along myosin II filaments [68, 69] and localizes near the *Z*-line and with mitochondria in cardiac muscle [70]. Tensin2 is structurally related to the focal adhesion-associated mechanosensory protein tensin1, which binds directly to both PP1α and integrin to regulate cell migration and adhesion [71–73]. Thus, PP1β or a PP1β-associated protein might regulate the previously observed Sgcg-mediated ERK1/2 signaling [18, 20, 24], directly or indirectly, as part of a larger signaling complex.

Other candidate sarcoglycan interactors in Fig. 3B are also mechanistically plausible. While many of these candidate interactors are not an integral part of the sarcolemma, per se, they demonstrate the utility of the enrichment strategy, as they represent proteins that bridge the sarcolemma with other cellular compartments, and sites where loss of function mutations cause neuromuscular diseases. For example, mutations in myotilin and titin are causal for LGMD [50], and Sgcg binds titin sequences [25]. Palladin scaffolds the formation of cytoskeletal structures, including costameres [74, 75]. Others of these proteins are involved in membrane trafficking (Ep215, TACC2, Mapt), cellular responses to stress (Naca, Prdx3), and nuclear function (eIF4G, PURA, nucleolin).

The extractabilities of PP1β, MYPT2, and NKCC1 into Rx and RIPA buffers were roughly comparable across genotypes (Additional file 6, Fig. S3). There was no apparent correlation between the amounts of these novel candidate interactors in the RIPA extracts (Additional file 6, Fig. S3) and the numbers of normalized spectral counts obtained after co-IP with anti-Sgcg from muscle (Fig. 3), especially for glycosylated and nonglycosylated NKCC1 [76, 77] (Additional file 6, Fig. S3C). These results suggest that neither large changes in protein expression levels nor extractability account for the observed reductions in the numbers of spectral counts in co-IPs from *Sgcg*^*−/−*^ muscle and that the reduction of these proteins in IP is due to the loss of a direct or indirect interaction involving Sgcg.

We used immunofluorescence co-localization in human rhabdomyosarcoma cells (RH30) as a secondary screen to interrogate potential interactions of the SC with PP1β and the PP1β-interacting proteins in a cell type that lacks high-level muscle protein organization. The rationale was that signal proximity in the absence of large assemblages of muscle proteins would be consistent with an association requiring only a few proteins. We used cDNAs encoding tagged NKCC1, PP1β, MYPT2, and tensin2 co-expressed with either untagged Sgcg or Sgcg-Flag, with and without co-expression of Sgcb-Flag and Sgcd-Flag; together these three sarcoglycans form the SC core [51, 64]. We also interrogated protein localizations with antibodies, as available, including two different, specific Sgcg antibodies (Additional files 7, 8, and 9; Figs. S4, S5, and S6).

### NKCC1 is an Sgcg interactor

**Fig. 4:**
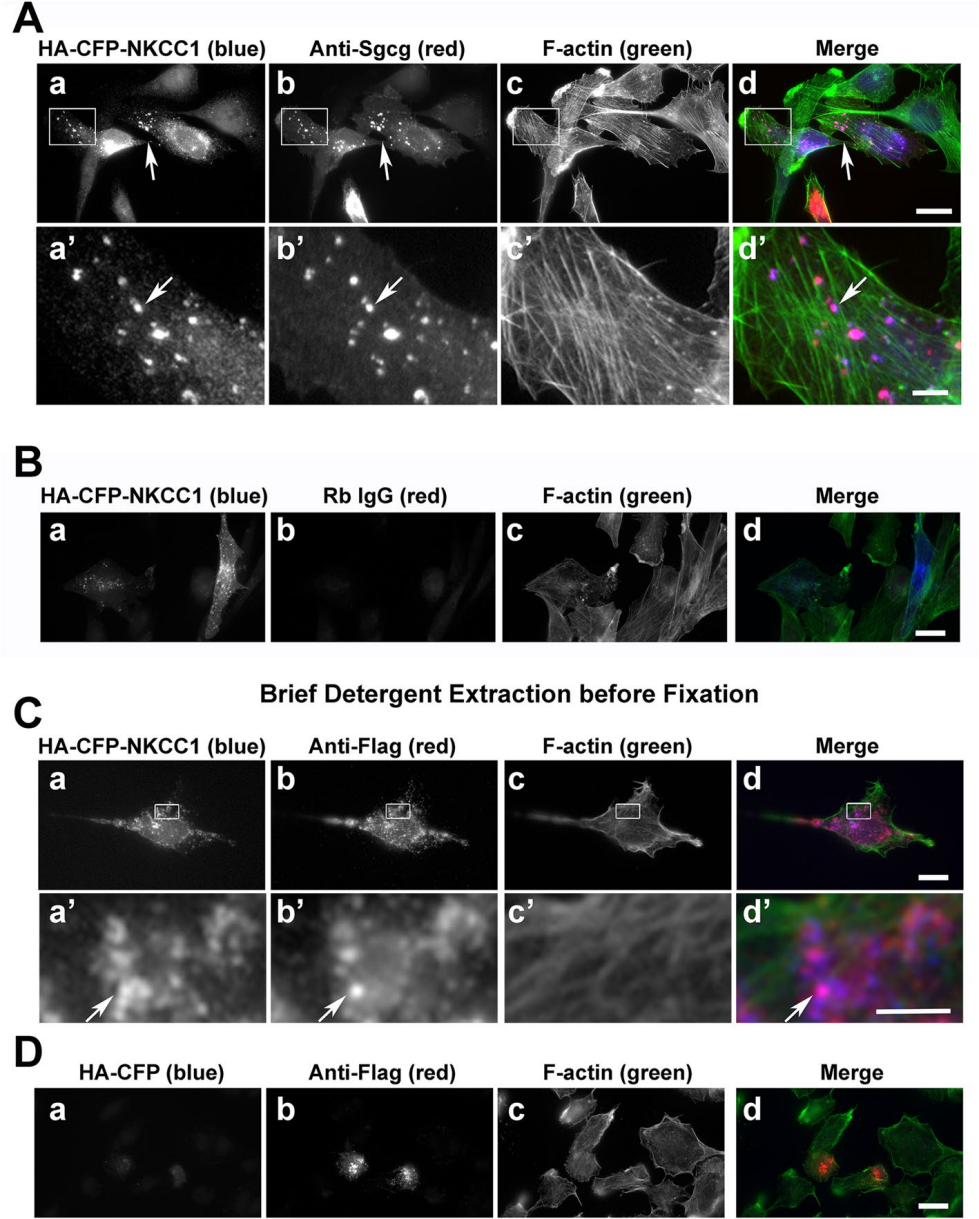
NKCC1 co-localizes with the sarcoglycan complex in large, detergent-stable vesicular structures. **A**, **B** HA-CFP-tagged NKCC1 (panels a, a’, blue in merges) co-localizes in punctae visualized with both **A** our affinity-purified anti-Sgcg and **B** anti-Sgcg from Proteintech Group (PTG) (panels b, b’, red in merges) after expression in RH30 rhabdomyosarcoma cells with Flag-tagged Sgcg, Sgcd, and Sgcb. Enlargements shown (a’–d’) of the boxed areas in panels a–d. **C** Specificity control with nonimmune rabbit (Rb) IgG substituted for anti-Sgcg. **D** Signal overlaps of HA-CFP-NKCC1 (panels a, a’, blue in merges) and the sarcoglycan complex (panels a, a’, red in merges) persist even when cells are briefly extracted with 0.1% Triton X-100 before fixation. **E** The HA-CFP tag alone (panel a, blue in merge) does not persist in pre-extracted RH30 cells co-transfected with Flag-tagged Sgcg, Sgcd, and Sgcb (panel a, red in merge). **A**–**E** Phalloidin-stained actin filaments delineate cell boundaries (panels c, c’, green in merges). Bars (d), 20 μm; bars (d’), 5 μm. Overlapping HA and Flag signals in magenta (arrows)

The novel candidate interactor with the strongest co-localization with co-expressed Sgcg in RH30 cells was NKCC1 (Fig. 4). RH30 cells express reduced levels of skeletal muscle proteins, supporting assessments of direct or indirect protein-protein interactions independent of the presence of an organized contractile apparatus [78]. Positive co-localizations of NKCC1 with Flag-tagged SC proteins were confirmed using Sgcg antibody (Fig. 4A, arrows, vs. B) and anti-Flag (Fig. 4C). Most co-localizations were seen in punctate structures suggestive of intracellular vesicles. To test whether these overlaps were due solely to co-existence of these membrane proteins in the same lipid bilayer, we briefly extracted RH30 cells expressing both proteins with detergent before fixation and staining. Under these conditions, the cytosolic control HA-CFP diffused away (Fig. 4D), but the signals from NKCC1 and sarcoglycans remained, as did the pronounced signal overlaps (Fig. 4C, magenta).

**Fig. 5:**
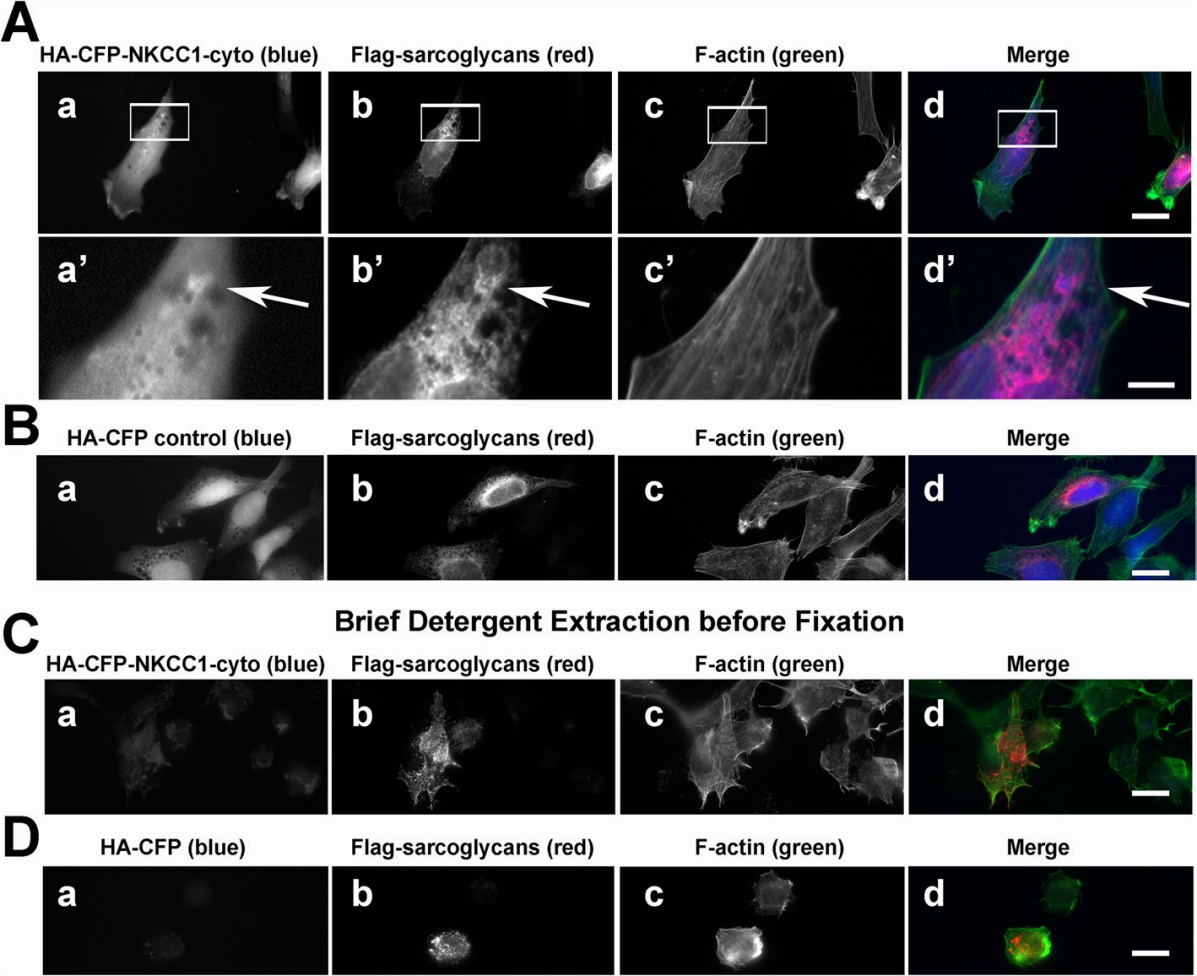
HA-CFP-NKCC1-cytoplasmic domains appear to be loosely associated with Flag-tagged SC proteins. RH30 cells were co-transfected in parallel with Flag-tagged sarcoglycans and stained as in Fig. 4. **A** HA-CFP-NKCC1-cytoplasmic domains and **B** HA-CFP (negative control; panels a, a’; blue in merges) were visualized with Flag-tagged sarcoglycans (panels b, b’; red in merges) and F-actin (panels c, c’; green in merges). After pre-extraction with 0.1% Triton X-100, **C** signal from residual HA-CFP-NKCC1-cytoplasmic domain was indistinguishable from **D** residual HA-CFP. Overlapping HA and Flag signals in magenta (arrows). Bars (d), 20 μm; bar (d’), 5 μm

Less pronounced signal overlaps in transfected RH30 cells were observed for the sarcoglycans and a fusion protein of HA-CFP with a truncated NKCC1 protein containing the cytosolic N-terminus directly fused with the cytosolic C-terminus (NKCC1-cyto); this NKCC1 truncation lacks the 12 transmembrane domains and extracellular sequences [56] (Fig. 5). Unlike full-length NKCC1 (Fig. 4A), this NKCC1-cyto protein was predominantly cytosolic (Fig. 5A); the cytosolic signal was intensified in the vicinity of sarcoglycan punctae (Fig. 5A, arrows). More of the NKCC1-cyto protein was located in the cytosol than was observed for the more nuclear HA-CFP control protein (Fig. 5B). Both the NKCC1-cyto (Fig. 5C) and the HA-CFP (Fig. 5D) proteins diffused away from the sarcoglycan punctae in cells pre-extracted with detergent, suggesting that any association between the SC and the NKCC1 cytoplasmic domains must be of low avidity.

By contrast, we observed no co-localization of sarcoglycans with tagged tensin 2 at focal adhesions (Additional file 10, Fig. S7) and limited signal overlap with MYPT2 (Additional file 11, Fig. S8) or PP1β (Additional file 12, Fig. S9). MYPT2 localized primarily with phalloidin-stained actin filaments (Additional file 11, Fig. S8), suggestive of an interaction with myosin II in stress fibers [79]. In some cells, MYPT2 overlapped with brightly stained juxtanuclear Sgcg punctae, but no associations with large Sgcg-containing punctae in the cell periphery were observed (Additional file 11, Fig. S8). Similarly, signals from co-expressed Sgcg and PP1β tagged at either end with EGFP were not clearly different from the EGFP control (Additional file 12, Fig. S9), even under hypoosmotic conditions that induce translocation of PP1α from the nucleus to the cytoplasm [80]. These negative results do not eliminate the possibility of an interaction with Sgcg, but suggest that any binding to PP1β or MYPT2 requires either post-translational modifications or muscle-specific interaction partners.

### Sgcg interacts with NKCC1 cytoplasmic domains

**Fig. 6:**
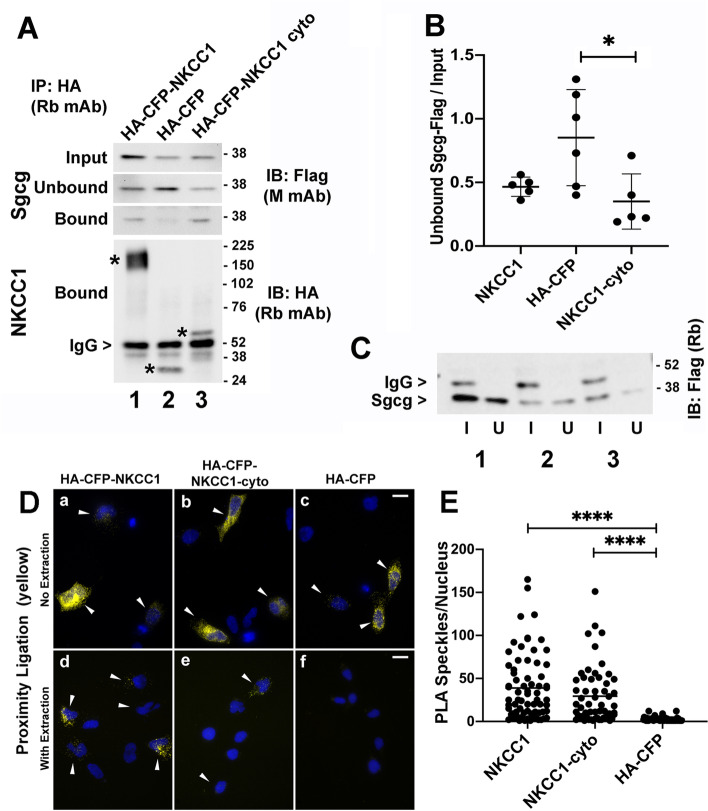
Flag-tagged Sgcg directly or indirectly associates with HA-CFP-tagged NKCC1 proteins. **A** Immunoblot of the most successful co-IP, showing more Sgcg-Flag sedimenting with the NKCC1 and NKCC1-cyto (bound), compared to the HA-CFP negative control; corresponding decreases were observed in supernatants after sedimentation (Unbound). Sgcg-Flag amounts in cell lysates also are shown (Input). Flag-tagged Sgcg, Sgcb, and Sgcd proteins were co-expressed with either HA-CFP-NKCC1 (lane 1), HA-CFP (lane 2), or HA-CFP-NKCC1 cytoplasmic domains (NKCC1-cyto, lane 3) in RH-30 cells and immunoprecipitated with anti-HA. Bound NKCC1 proteins were visualized with anti-HA (asterisks); bead-bound rabbit anti-HA heavy chain (IgG). **B** Densitometric quantification of Unbound/Input ratios of Sgcg-Flag from supernatant-depletion assays, such as those shown in **C**, and analysis by one-way ANOVA with Tukey’s multiple comparisons test. *P* values for the mean differences between HA-CFP control and HA-CFP-NKCC1 and HA-CFP-NKCC1-cyto were 0.12 and 0.02, respectively (*P < 0.05). Central bars, means; error bars, S.D. **C** Representative immunoblot for supernatant-depletion assays in **B**, showing decreases in band intensity in Unbound lanes (U), compared to Inputs (I). IP used rabbit monoclonal anti-HA (HA); Sgcg-Flag was visualized with rabbit anti-Flag (Flag); HA-CFP-tagged NKCC1 (lane 1), HA-CFP alone (lane 2), or HA-CFP-NKCC1 cytoplasmic domains (lane 3). **D**, **E** Representative images and quantification of PLA for the SC interaction with HA-CFP-NKCC1, HA-CFP-NKCC1-cyto, or the HA-CFP control in RH30 cells co-transfected with Flag-tagged Sgcg, Sgcb, and Sgcd. **D** In cells fixed without detergent pre-treatment to remove free cytosolic proteins (Figs. 4D and 5D), PLA speckles indicating proximity of anti-Sgcg and anti-HA were observed with all three HA-CFP constructs (**D**a–c). A brief detergent pre-extraction revealed persisting proximity of the SC complex with NKCC1 and NKCC1-cyto, but not with the HA-CFP tag alone (**D**d–f, **E**). PLA speckles are pseudocolored yellow; DAPI-labeled nuclei in blue. Size bars, 20 μm. **E** Graph of PLA speckles per nucleus for pre-extracted cells. Cells with no PLA speckles were assumed to be untransfected and were not included. Horizontal bars, means from results combined from 2 experiments with the following total numbers of counted cells: NKCC1 (70), NKCC1-cyto (56), HA-CFP (65). Transfection efficiencies varied from 36% to 65%, with higher values for HA-CFP, despite a lower percentage of PLA speckles. ****P < 0.0001; P for NKCC1 versus NKCC1-cyto was 0.6140 (ns), from a one-way ANOVA with a Kruskal-Wallis multiple comparisons test

To further examine the interactions of the SC with NKCC1 and the NKCC1-cyto domains, we co-overexpressed the tagged proteins in RH-30 cells and used the tags for co-IP, supernatant-depletion assays, and PLA (Fig. 6). Initial co-IP experiments using brief, low-stringency washes revealed that more HA-CFP-NKCC1 and NKCC1-cyto protein than HA-CFP negative control co-sedimented with Flag-tagged sarcoglycans (Sgcb, Sgcd, Sgcg) (Fig. 6A). However, signal was lost if cell lysates were subjected to freeze-thaw or if the IP pellets were washed stringently, suggesting that any interaction was of low avidity *in vitro*. We therefore pursued supernatant-depletion assays for better quantification of low-avidity interactions [61]. In these assays, the amount of the Unbound partner protein in the supernatant is monitored before and after sedimentation of the bound protein to quantify the initial binding equilibrium [61]. The Unbound-to-Input ratio reveals the amount of target protein lost with the bound pellet, without a requirement that the interaction withstands the dilutions and time required for rinsing. Ratios less than 1 are consistent with a binding interaction. We consistently found Unbound/Input ratios less than one for Sgcg-Flag sedimented by full-length HA-CFP-NKCC1 or HA-CFP-NKCC1-cyto, as compared to the HA-CFP control protein, although only the result with HA-CFP-NKCC1-cyto was statistically significant due to the variability of the control (Fig. 6B, C). The only observed Flag-associated signal was from Sgcg-Flag, possibly due to the presence of endogenous Sgcb and Sgcd in these cells (https://www.proteinatlas.org). By contrast, supernatant-depletion assays with co-expressed Flag-tagged sarcoglycans and PP1β tagged with EGFP at either end resulted in no ratios of Unbound to Input less than 1 in co-IP experiments that used either anti-Flag or anti-EGFP. PLA of transfected cells further supported the existence of a direct or indirect interaction between the SC and NKCC1 (Fig. 6D, E). In unextracted co-transfected cells, all HA-CFP tagged constructs showed proximity with the SC (Fig. 6D, top row). After pre-extraction with low levels of detergent to eliminate free cytosolic proteins, significantly more PLA signal was observed for the SC with NKCC1 and NKCC1-cyto than with the HA-CFP negative control (Fig. 6D, E). These results support the existence of a low-avidity association between exogenously expressed sarcoglycans and the NKCC1 cytoplasmic domains.

### NKCC1 co-localizes with the DGC and affects Sgcg-dependent ERK activation in muscle

**Fig. 7:**
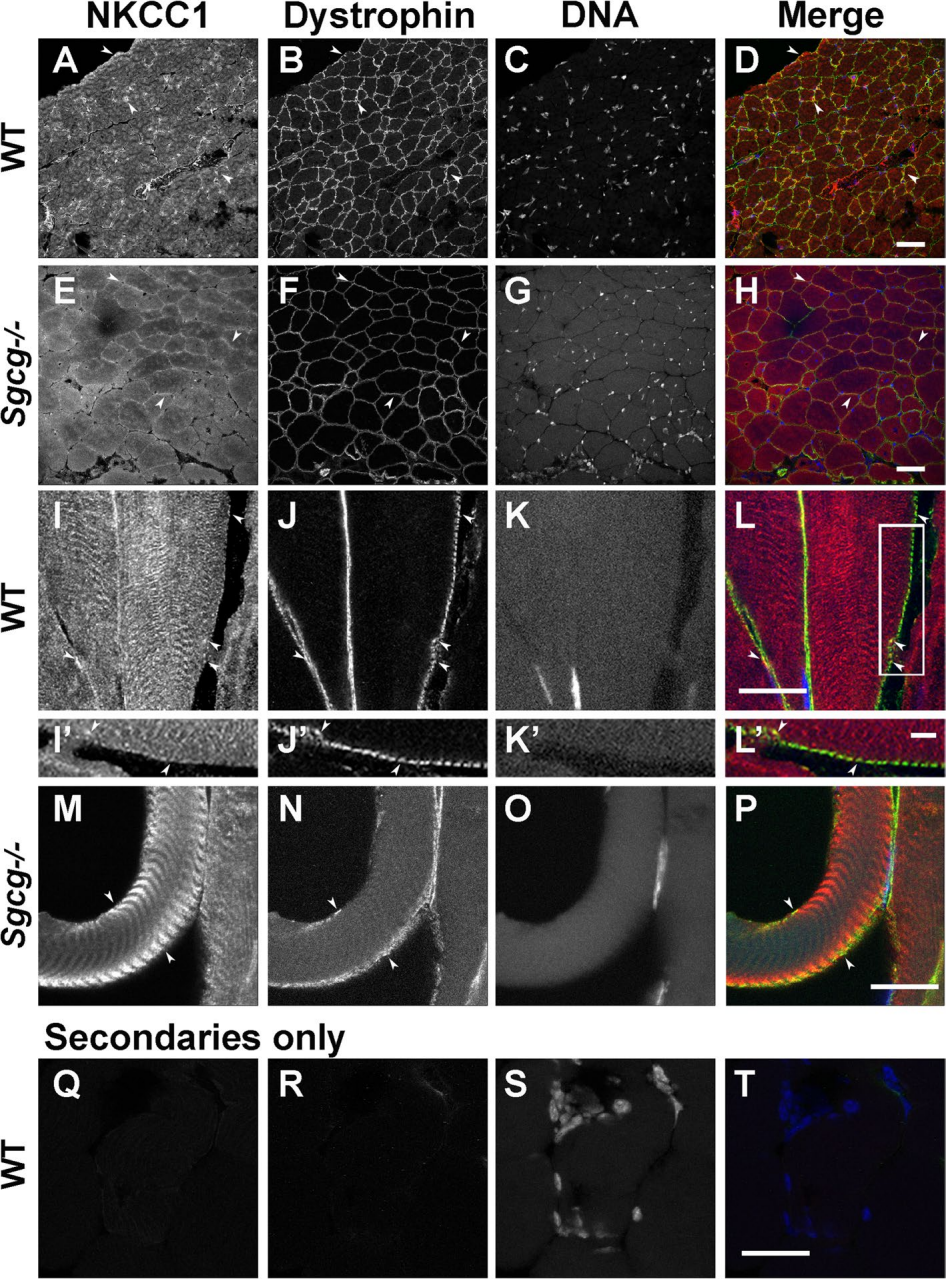
Confocal microscopy of NKCC1 and dystrophin in EDL muscles from WT and *Sgcg*^−*/*−^ mice. **A**–**H** Cross sections show overlapping signals at the sarcolemma (arrowheads; yellow in merges), as well as internal myofibrillar NKCC1 staining in both genotypes. **I**–**P** In longitudinal sections, the internal staining suggests an association with T-tubules, which end at dystrophin-associated costameres in the sarcolemma [8, 9, 82]. **I’**–**L’** Enlargements of the area boxed in **L**. **Q**–**T** Sections stained with only secondary antibodies. Bars are **A**–**H** 50 μm; **I**–**T** 20 μm; **I’**–**L’** 5 μm

To see if NKCC1 localizes in the vicinity of the SC and thus might associate *in vivo*, we localized NKCC1 in murine EDL muscles (Fig. 7). NKCC1 was known to be in skeletal muscle fibers [81], but its localization with respect to dystrophin at costameres in muscle fibers [8, 9] was unexplored. In immunostained muscle cryosections, the anti-NKCC1 signal exhibits the same distribution as that of sarcolemmal dystrophin in both the presence of Sgcg in control muscle (Fig. 7A, B, I, J) and in its absence in muscles from *Sgcg*^*−/−*^ mice (Fig. 7E, F, M, N). Additional NKCC1 signal is associated with internal striations (Fig. 7I, M), consistent with localization at or near the T-tubule network that synchronizes calcium release within the muscle, and appears to be concentrated in the vicinity of T-tubule mouths at costameres [82, 83]. The similar signal distributions in the presence and absence of the SC indicate that NKCC1 does not require Sgcg for expression and transport to the sarcolemma. These results place NKCC1 into proximity with the DGC in vivo.

**Fig. 8:**
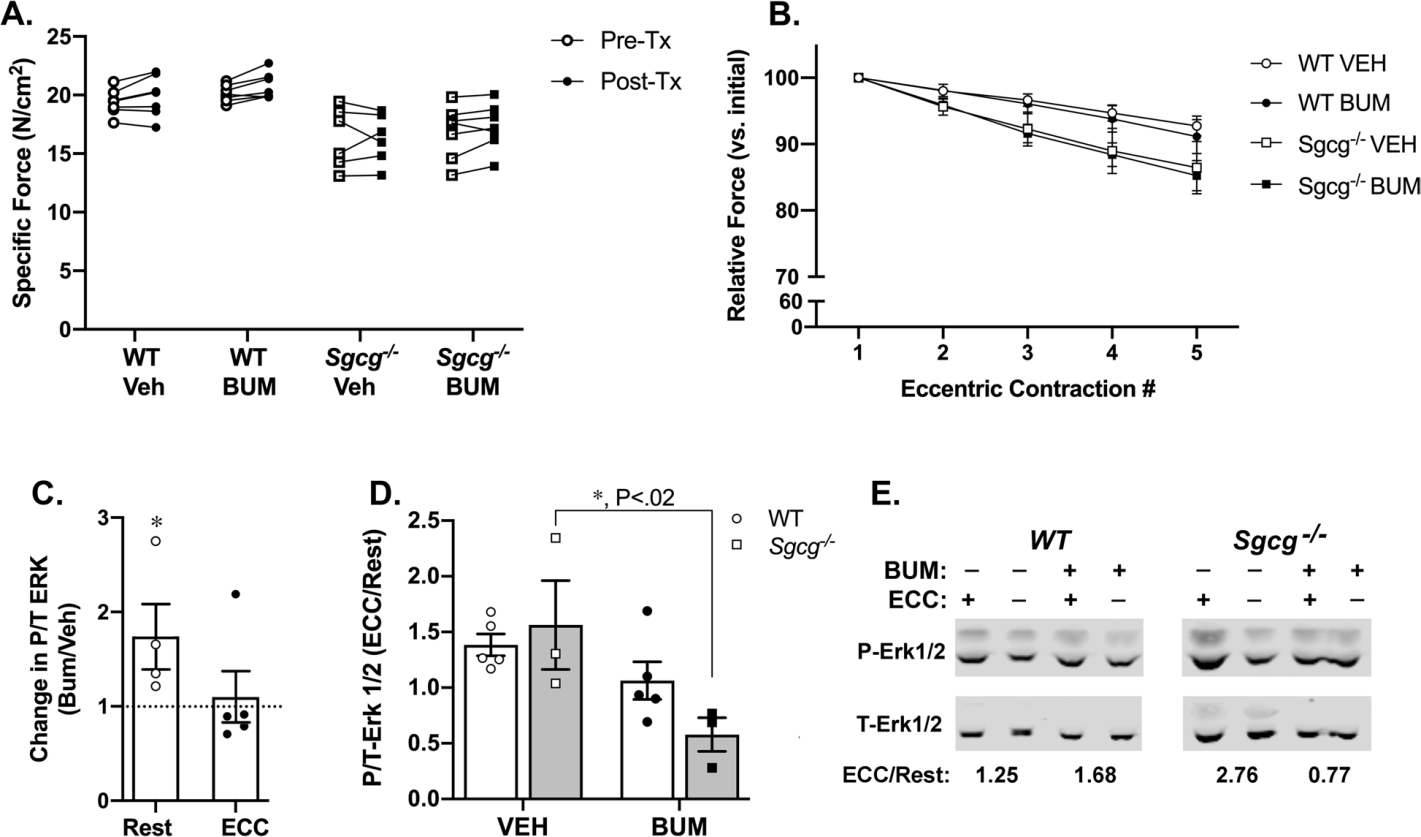
Effects of NKCC1 inhibition on muscle force generation and ERK phosphorylation. **A** Isometric tetanic force generation of EDL muscles is not altered by bumetanide (BUM) incubation, as compared with vehicle (VEH) control. Raw values showing forces in Ringer’s solution as pre-treatment (Pre-Tx), and the forces following 20–30-min incubation in 50 μM bumetanide or vehicle (ethanol) as post-treatment (Post-Tx). **B** Decrement of force following eccentric contractions (ECC) is not affected by bumetanide incubation. Forces are relative to the initial force in the first ECC. Means shown; error bars SEM. **C** levels of phosphorylated ERK are elevated in resting EDL muscles from wild-type mice, but the P-ERK response to ECC is unaffected. (*, *P* < 0.05, paired t-test between bumetanide and vehicle incubation for muscles from the same mouse). **D** Bumetanide incubation inhibits ECC-induced increases in P/T-ERK in the absence of Sgcg. *N* = 3–6 muscles per condition. *Significant difference between vehicle and bumetanide within strain (*P* = 0.0196), by 2-way ANOVA followed by Sidak’s multiple comparisons test. **C**, **D** Central lines, means; error bars, SEM. **E** Representative immunoblots. The signals from phosphorylated (P) to total (T) ERK signal were determined for each condition, and then the ratio of the P/T ERK signals after ECC were divided by the corresponding ratio at rest for each condition (ECC/Rest)

We also explored whether NKCC1 co-transporter activity affects SC-mediated ERK signaling [18, 24]. NKCC1 co-transporter activity increases K^+^-induced calcium transients in muscle by facilitating the influx of the chloride counter ion [84]. We found that NKCC1 activity also alters P-ERK responses. The NKCC1 co-transport inhibitor bumetanide had no significant effect on the specific forces of EDL muscle contraction or the relative loss of force following a series of eccentric contractions (Fig. 8A, B). However, increased P-ERK was evident in resting WT muscle incubated in bumetanide (Fig. 8C). After eccentric contractions (ECC), bumetanide inhibited ERK phosphorylation in *Sgcg*^*−/−*^ muscles, but not in WT muscle (Fig. 8D, E). Sgcg-dependent changes in total glycosylated NKCC1 and in NKCC1 phosphorylation, a measure of NKCC1 activation [66, 85, 86], might occur during ECC strain, but the differences were not statistically significant (Additional file 13; Fig. S10). These results support a role for NKCC1 in Sgcg-dependent ERK activation in response to ECC [18], without a Sgcg-dependent alteration of NKCC1 phosphorylation.

### New candidate Sgcg interactors link the SC with survival signaling pathways in silico

**Fig. 9:**
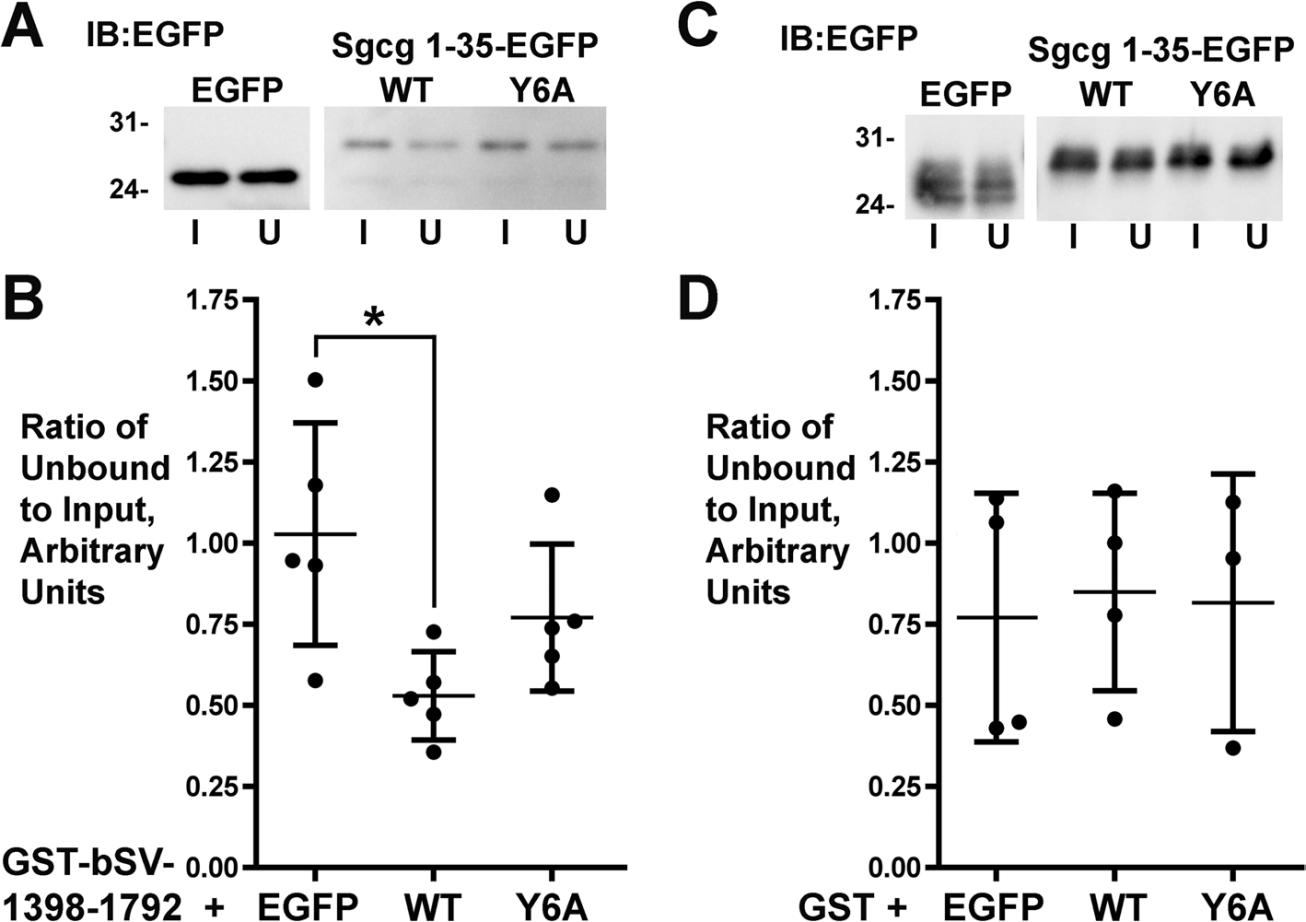
Direct binding of recombinant Sgcg N-terminus to the conserved archvillin/supervillin C-terminus. Supernatant depletion assays showed a significant loss of recombinant Sgcg-1-35-EGFP, but not of recombinant EGFP, after pull down with **A**, **B** GST-tagged bovine supervillin (bSV) residues 1398-1792 or **C**, **D** GST alone. Mutation of tyrosine-6 in Sgcg to alanine (Y6A) may affect this interaction. **A**, **C** Representative immunoblots of Input (I) and Unbound (U) fractions. **B**, **D** Densitometry quantification of replicate experiments. Statistical analyses employed one-way ANOVA, with a Tukey-Cramer multiple comparisons post-test. *N* = 3–5, as shown; *P < 0.05. Central lines, means; error bars, S.D.

We also used in vitro assays to evaluate the directness of the interaction between Sgcg and archvillin, a membrane-cytoskeleton linker protein that is a scaffold for ERK1/2 signaling [27, 30]. This interaction was originally identified with yeast two-hybrid assays and supported by co-IP [26]. Because initial co-IPs suggested a low-avidity interaction similar to that observed for Sgcg and NKCC1 proteins, we carried out supernatant-depletion assays with purified, recombinant Sgcg N-terminus (Sgcg 1-35-EGFP, WT) and the conserved archvillin/supervillin C-terminus (Fig. 9). Soluble EGFP and Sgcg 1-35-EGFP proteins were purified and used with purified GST-tagged archvillin C-terminus (Fig. 9A, B) or GST alone (Fig. 9C, D) in GST-pulldown assays. We observed a statistically significant direct interaction between the archvillin C-terminus and WT Sgcg (Fig. 9A, B). In addition, Sgcg 1-35-EGFP with a mutation in Tyr-6 (Y6A), the residue implicated in P-ERK signaling in muscle [24], exhibited a mean Unbound/Input ratio that was intermediate between that observed with Sgcg WT and the EGFP control, but was not statistically significant from either value. No differences among the proteins were observed with the GST control beads (Fig. 9C, D). These results support the directness of the previously described Sgcg interaction with archvillin [26] and suggest that Sgcg-Y6 may be involved in the Sgcg-archvillin interaction.

**Fig. 10:**
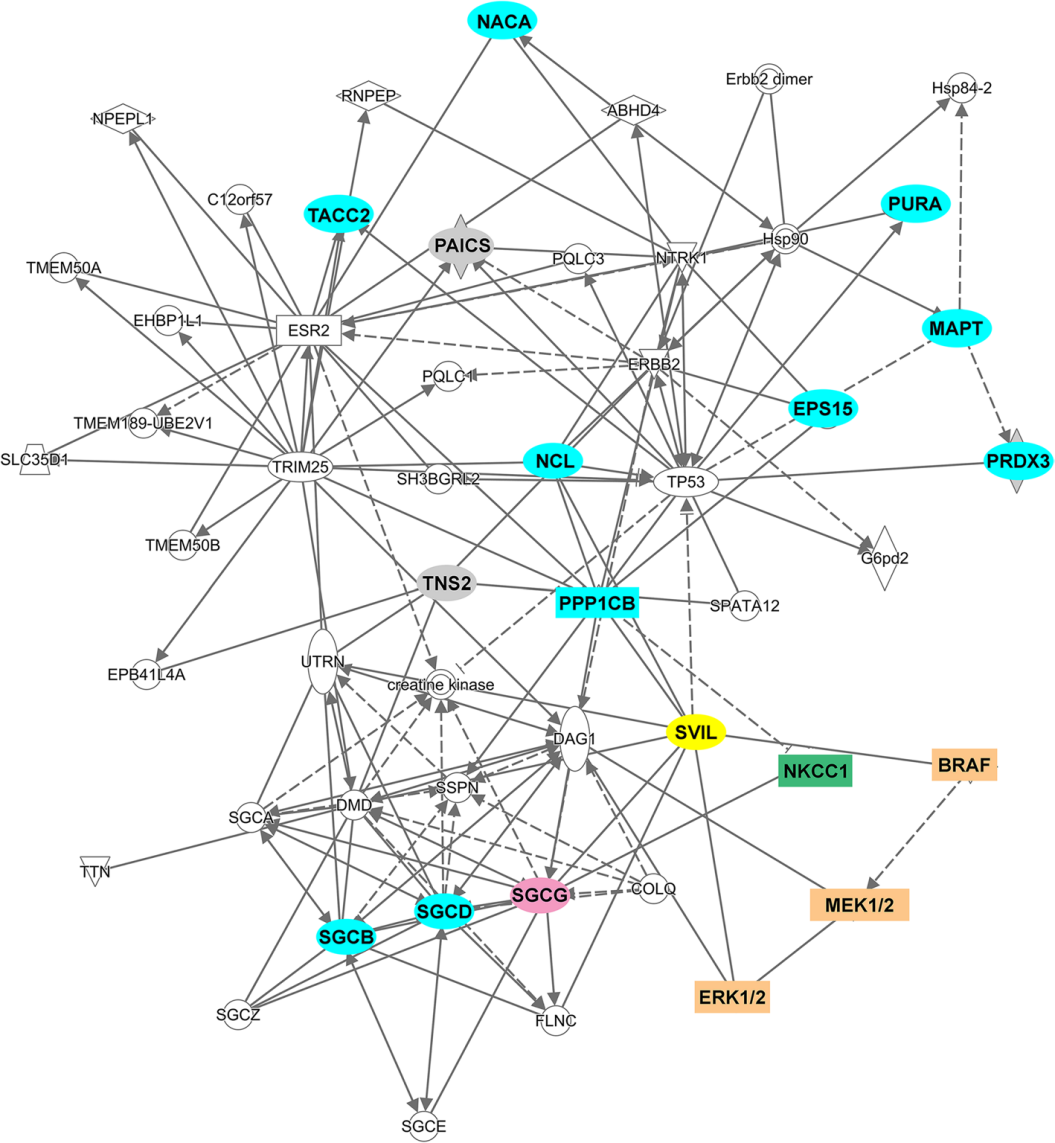
Pathway analysis suggests cross-talk between interconnected survival signaling pathways and sarcolemmal sarcoglycans, dystrophin, and archvillin. The gene names of proteins co-immunoprecipitating with or binding to Sgcg as shown here were arranged in the default Organic View using IPA software. Eleven of 13 Top Candidate Sgcg interactors, as well as 3 of 5 Other Candidate interactors (Fig. 3), were assigned to either the TP53/ESR2/TRIM25-associated signaling network or to the sarcoglycan, dystrophin, dystroglycan-associated sarcolemmal network. Colors highlight SGCG (magenta), SVIL (yellow), NKCC1/SLC12A2 (green), additional Top Candidate Interactors (blue) and Other Candidate Interactors (gray). Additional genes (white) and direct (solid lines) and indirect (dashed lines) interactions were added by the IPA software. Interactions involving the B-Raf, MEK1/2, and ERK1/2 signaling cascade (orange) with β-dystroglycan/DAG [88] and SVIL [30] were added manually

The Sgcg-archvillin direct interaction and the new candidate direct or indirect interactors reported in Fig. 3 were then analyzed using IPA software to graphically integrate the new associations described here with published data [87] (Fig. 10). Most candidate proteins fell into one of two pathways: (1) the dystrophin- and sarcoglycan-associated DGC or (2) an interconnected signaling pathway containing p53 (gene name *TP53*), estrogen receptor β (*ESR2*), and the ubiquitin E3 ligase TRIM25. Eleven of the 13 top candidate interactors (Figs. 3 and 10, blue), including NKCC1 (gene *Slc12a2*, green), archvillin (*Svil*, yellow), and 3 of the 5 other candidate interactors (Fig. 10, gray) identified in Sgcg IPs (Fig. 3), were assigned by the program as components of this survival signaling pathway or the sarcolemmal network. With the addition of the Sgcg-archvillin interaction [26] (Fig. 9) and the new Sgcg candidate interactors (Fig. 3), the software readily merged the sarcolemmal and signaling pathways into a single larger network (Fig. 10). We then manually added additional links to show the published scaffolding of ERK and the ERK-activating kinase MEK2 by β-dystroglycan (gene *DAG*) [88], the scaffolding of ERK and the MEK1/2-activating kinase B-Raf (gene *BRAF*) by the smooth muscle isoform of archvillin (gene *SVIL*, Fig. 10, yellow) [30], and the interaction demonstrated here between Sgcg (Fig. 10, pink) and NKCC1 (gene *SLC12A2*) (Figs. 4, 5, and 6). The merger of these interactors into a single network in silico is consistent with a role for sarcoglycan interactors in ERK regulation and survival signaling in muscle.

## Discussion

Using a proteomics approach optimized for recovery of muscle membrane proteins, we identified novel candidate direct or indirect interactors for Sgcg, and by extension, the SC. Our screen identified all subunits of the SC, plus 16 other proteins that specifically co-IP with a high-avidity Sgcg antibody from muscles that contain the SC but are reduced in muscles from *Sgcg*^*−/−*^ mice, which lack these proteins (Fig. 3). Differences in our approach versus those in previous studies include prior enrichment for membranes and the analysis of cytoskeleton-associated, detergent-resistant membranes using an IP buffer with SDS. The use of *Sgcg*^*−/−*^ muscles as a specificity control eliminates proteins present in the co-IPs due to low-avidity antibody cross-reactivities not recognizable by immunoblots or IF (Additional files 3 and 4; Tables S2 and S3). It is especially noteworthy that most of the identified top and other candidate interactors in Fig. 3 were readily grouped in silico into a single signaling pathway that links ERK regulation with survival signaling (Fig. 10).

NKCC1 emerged as a potential interactor with Sgcg in a secondary screen, which consisted of co-localizing exogenously expressed SC proteins and candidate interactors (Fig. 4, Additional files 10, 11, and 12, Figs. S7, S8, and S9). Immunofluorescence co-localization (Fig. 5), co-IP (Fig. 6A), supernatant-depletion assays (Fig. 6B, C), and PLA (Fig. 6D, E) were consistent with a low-avidity direct or indirect interaction between the SC and the NKCC1 cytoplasmic domains that may be comparable in magnitude to the direct, low-avidity binding of the Sgcg N-terminus to the archvillin C-terminus (Fig. 9). We also show that NKCC1 overlaps with dystrophin at skeletal muscle costameres and with internal muscle striations (Fig. 7), sites that are likely T-tubules involved in intracellular Ca^++^ regulation [82, 89]. Physiological analyses with the NKCC1 inhibitor bumetanide also suggest a role for Na^+^K^+^2Cl^−^ co-transport during SC-mediated ERK signaling in skeletal muscle (Fig. 8). The NKCC1 localization in proximity to T-tubules and the apparent role of NKCC1 co-transport in sarcoglycan-regulated signaling after ECC strain support a direct or indirect interaction of NKCC1 with the SC.

The high-avidity association observed between full-length NKCC1 and Sgcg during co-IP in the SDS-containing RIPA1 buffer (Fig. 3) and after pre-extraction with detergent in IF (Fig. 4) suggests that this association involves more than the NKCC1 cytoplasmic domains used in our in vitro assays (Fig. 6). Interactions observed in supernatant-depletion assays or PLA, such as those of Sgcg with the NKCCI cytoplasmic domains (Fig. 6) and with archvillin (Fig. 9), may be of relatively low avidity. A low-avidity interaction between Sgcg and archvillin is consistent with its identification in yeast two-hybrid assays [26], which also detect weak interactions [90]. Low-avidity interactions are often cumulative, regulated by post-translational modifications, and known to play important roles in signaling networks [91, 92]. Other interactions involving the NKCC1 transmembrane or extracellular domains are likely required for a tight association with Sgcg in vivo, either through direct binding or by indirect associations involving other proteins in this emerging signaling network. Transmembrane domains, by themselves, can enhance the avidity of an association ~100 fold by orienting and concentrating both partners at the membrane surface [93]. Multiple connection(s) between NKCC1 and the DGC also are suggested by the SC-independent localization of NKCC1 in *Sgcg*^*−/−*^ muscles (Fig. 7). Thus, the DGC-associated signaling network may consist of many post-transcriptionally regulated interactions with variable and regulatable avidities.

Although the results from the secondary screens involving PP1β, MYPT2, and tensin did not support an interaction with the SC, these results do not eliminate the possibility of indirect or conditional associations. For instance, PP1β may link indirectly to Sgcg through binding to NKCC1 [66]. Furthermore, assays with PP1β in cell lysates are challenged by the presence of the ~ 200 known PP1β-binding proteins that could act as endogenous competitive inhibitors [94]. Regulation of PP1β localization or function at costameres by Sgcg is still possible, given the cross-talk between the SC and P-ERK signaling [18, 20, 24].

Our novel approach has expanded the list of candidate SC-interacting proteins and thus complement results from previous studies [95, 96]. Most of our candidate interactors were obtained from a cytoskeleton-associated membrane fraction that has been typically not interrogated. Also, the inclusion of SDS in the RIPA1 buffer may have disrupted many previously reported interactions. For instance, β-dystroglycan is a well-accepted interactor of the SC [5, 97] that is present in our RIPA1 lysate (Fig. 2E), but did not appear as a specific interactor in our co-IPs (Fig. 3). The accepted β-dystroglycan-SC interaction emerged from co-IPs with nonionic detergent extracts of membranes not tightly bound to the cytoskeleton [95, 96]. The procedural differences may explain the absence of β-dystroglycan and other published SC-associated proteins from our list of candidate interactors.

A role for NKCC1 in skeletal muscle mechanotransduction is consistent with prior studies. NKCC1 has long been appreciated as necessary for muscle volume control, which is regulated by P-ERK under osmotic, but not hyperosmotic, conditions [98]. NKCC1 is implicated in myoblast differentiation and exercise-induced hypertrophy of skeletal muscle [84], as well as in phenylephrine-mediated rhythmic contractions of smooth muscle [99]. NKCC1 activity also promotes transient decreases in muscle force in mouse models of hypokalemic periodic paralysis [100, 101]. The mechanism of action is thought to involve increased transient accumulations of intracellular calcium ions that are enhanced by NKCC1-dependent increases in the chloride counter-ion concentration subjacent to the sarcolemma [99, 102, 103]. ECC promotes Ca^++^ entry across the sarcolemma [104], and increased calcium influx induced by a number of genetic mechanisms is sufficient to induce muscular dystrophy [105, 106]. Thus, NKCC1 function at the SC can be plausibly linked to molecular events leading to long-term muscle damage.

Our physiological analyses suggest that NKCC1 transporter activity reduces ERK activation in resting wild-type muscle and promotes a SC-dependent increase in P-ERK after ECC. ERK1/2 activation protects myofibers from damage and contributes to cardiac hypertrophy and protein expression of dystrophin and utrophin in skeletal muscle [29, 107, 108]. NKCC1 transporter activity thus may be antagonistic to ERK activation in resting muscle and contributory to stimulus-induced ERK activation after ECC. In fact, prior studies have positioned NKCC1 activity both upstream and downstream of ERK activation. Inhibition of ERK signaling in tracheal epithelial cells reduced bumetanide-sensitive ^36^Cl^−^ uptake, a measure of NKCC1 activity [109]. However, NKCC1 inhibition or depletion attenuated P/T ERK phosphorylation in fibroblasts [110, 111], astrocytes [112], HeLa cells [113], and injured rat brains [114]. Epidermal growth factor stimulation of corneal epithelial cells promoted co-IP of NKCC1 and P-ERK1/2 [115], suggesting stimulus-mediated association. Up-regulation of P-ERK1/2 also is consistent with the anti-apoptotic activity of NKCC1 in cancer cells [116]. Thus, NKCC1 provides a route through which the SC could coordinate Ca^++^ and P-ERK signaling, with the differences between resting and stimulated muscle due to regulation of NKCC1 scaffolding of PP1β and signaling kinases [66]. Muscle strain also may affect coordination or competition between the NKCC1 and archvillin/kinase/PP1β scaffolds in binding to the SC.

The regions of signal overlap between NKCC1 and dystrophin in muscle imply proximity to costameres, the probable site of SC signaling. Costameres are structured lipid microdomains rich in caveolin-3, cholesterol, detergent-insoluble lipids, and cytoskeletal proteins and are a type of “lipid raft” [117]. Cholesterol-rich lipid rafts regulate many ion channels through altered conformations or associations with other proteins [118, 119]. Loss of caveolin-3 results in loss of costameric Sgca and enhanced ERK signaling [117]. NKCC1 co-fractionates from rat brain with lipid raft-associated proteins, and NKCC1 activity is inhibited by disruption of these domains in native membranes [120], suggesting that costameric lipid rafts up-regulate NKCC1 activity. Conversely, NKCC1 has been shown to interact with and inhibit the insulin-regulating, L-type amino acid transporter LAT1/SLC7A5 [121], leading to decreased ERK, Akt, and mTOR signaling [113]. Costameric NKCC1 therefore could regulate ERK signaling through conformational changes or shifts in localization of other costameric proteins.

Although the full mechanism of SC-mediated signaling remains to be elucidated, the candidate Sgcg interactors identified here suggest mechanisms for intervention in many myopathologies. The role of NKCC1 in promoting chloride uptake [67] may be mechanistically important because the resulting increases in cytosolic Cl^−^ could support the higher concentrations of intracellular Ca^++^ responsible for muscle damage by serving as Ca^++^ counterions [106]. The candidate interactors involved in estrogen-, TRIM25- and TP53-associated signaling pathways also suggest cross-talk with anti-apoptotic pathways [122] and additional avenues for further investigation, including potential relevance to NKCC1-based genetic disorders [123].

## Conclusions

Our results indicate that NKCC1 associates directly or indirectly with the sarcoglycan complex in mouse muscle. Inhibition of NKCC1 transport activity is consistent with a role in sarcoglycan-mediated regulation of ERK1/2 signaling after ECC. Pathway analysis of other candidate Sgcg interactors suggests survival signaling mechanisms that may be manipulatable clinical targets in limb girdle muscular dystrophy and other myopathologies.

## Supplementary Information

**Additional file 1: Table S1.** Primers used in this study.

**Additional file 2: Figure S1.** Validation of new and commercial polyclonal antibodies and expression of SC proteins in RH30 cells. New affinity-purified rabbit (Rb) antibodies were generated against murine archvillin (mAV) residues 121-568 (anti-mAV) and against Sgcg residues 72-290 (anti-Sgcg). Full-length immunoblots show that (A) anti-mAV and (B) our anti-Sgcg are specific for the expected ~245-kDa and ~35-kDa bands in mouse muscle, but extra bands are present in RH30 cell lysates. Specificity of the major bands is confirmed by their absence from muscles lacking the immunogen and by increased staining in transfected cells. (C) Staining is similar for Proteintech Group (PTG) anti-Sgcg antibody. Immunoblots of *gastrocnemius* muscle proteins from wild-type (WT, lanes 1) and *Svil*^*-/-*^ or *Sgcg*^*-/-*^ muscles (lanes 2), untransfected RH30 cells (lanes 3) and RH30 cells transfected with vectors encoding Sgcg- Flag (lanes 4), Sgcd- Flag (lanes 5) or Sgcb- Flag (lanes 6). Exogenous expression in RH30 cells was confirmed for Flag-tagged (B, C) Sgcg, (D) Sgcb and (E) Sgcd. No endogenous sarcoglycans were detected in RH30 cells. (B, C) The same blots used for anti-Sgcg staining were stripped and re-probed with anti-actin as a loading control. No cross-reactivity of anti-Sgcg was observed with the structurally similar Sgcd protein. Minor immunoreactive higher molecular mass bands at ~70 kDa and ~160 kDa in RH30 cells are variably present. These larger bands may represent SDS-resistant complexes of sarcoglycans [49, 63]. Positions of molecular mass markers in kDa are shown on the left. Arrows denote specific bands of the expected size.

**Additional file 3: Table S2.** Samples report for all proteins identified in immunoprecipitates from Rx buffer. Normalized weighted spectral counts from all immunoprecipitations performed in Rx buffer; dataset before manual curating, as described in Methods.

**Additional file 4: Table S3.** Samples report for all proteins identified in immunoprecipitates from RIPA1 buffer. Normalized weighted spectral counts from all immunoprecipitations performed in RIPA1 buffer; dataset before manual curating, as described in Methods.

**Additional file 5: Figure S2.** Phase contrast micrographs of *gastrocnemius* muscle membrane fractions. Fractions, referenced as described in Fig. 1A, from (A, D, G, J) C57BL/6 (WT), (B, E, H, K) *Sgcg*^*-/-*^ and (C, F, I, L) *Svil*^*-/-*^ mice were visualized by phase-contrast microscopy. Large membrane fragments (arrows) were observed in (A-C) total muscle extracts and (D-F) crude membrane pellets (Lane 4), but only small particulates were present in the supernatants after extraction with (G-I) Rx or (J-L) RIPA1 buffers. Bar, 0.2 mm.

**Additional file 6: Figure S3.** Fractionation of selected candidate Sgcg interactors from the different muscle genotypes. Fractions from ~100 mg skeletal muscle were extracted as shown in Fig. 1, immunoblotted and probed for the Sgcg candidate interactors PP1β (Life Span Biosciences), MYPT2 (Proteintech Group), or NKCC1 (Alomone Laboratories). All fractions are normalized as in Fig. 2. Each immunoblot is representative of 3 (*Sgcg-/-*, *Svil-/-*) or 4 (WT) independent biological replicates. The multiple bands for NKCC1 are a consequence of differential glycosylation [74, 75].

**Additional file 7: Figure S4.** Validation of rabbit (Rb) polyclonal antibodies against Sgcg for immunofluorescence microscopy. Evaluation of Proteintech Group Inc. (PTG, A-D) and our new affinity-purified (ours, E-H) anti-Sgcg antibodies for immunofluorescence microscopy, as compared to nonspecific Rb IgG (I-L), in RH30 cells transfected with plasmids encoding Sgcg-Flag. Both anti-Sgcg antibodies specifically recognize structures stained with anti-Flag (arrows). Overlapping red and green signals appear as yellow.

**Additional file 8: Figure S5.** Validation of rabbit (Rb) polyclonal antibodies against NKCC1. (A, C) Full-length immunoblots of mouse muscle and RH30 cells with and without transfected HA-CFP-NKCC1; (B, D) Anti-NKCC1 (panels a, d; red in merges) and murine (M) anti-HA staining (panels b, e; blue in merges) in RH30 cells transfected with HA-CFP-NKCC1. Anti-NKCC1 antibodies were from (A, B) Alomone Labs, #ANT-071 and (C, D) Proteintech Group, #13884-1-AP. Both anti-NKCC1 antibodies recognized the expected bands in the 97-220 kDa molecular mass range (A, C). Similar staining patterns have been reported previously, with the multiplicity of bands attributed to differential splicing and variations in glycosylation [74, 75]. In immunofluorescence (B, D), only the Proteintech antibody (Da, Dd) exhibited enhanced staining in RH30 cells expressing high levels of exogenous HA-CFP-NKCC1 (B, D, arrows). Overlapping signals appear as magenta in Merges (c and f). Bars, 20 μm.

**Additional file 9: Figure S6.** IF signal of AbCam rabbit monoclonal anti- PP1β correlates with EGFP signal from EGFP-PP1β or PP1β-EGFP. (A) Full-length immunoblots of mouse muscle and RH30 cells with and without transfected PP1β-EGFP, as indicated; lanes 1 and 5 show molecular mass markers. (B) Anti-PP1β (panels a, e; red in merges) and EGFP fluorescence (panels b, f; green in merges) in RH30 cells transfected with PP1β cDNA, as indicated. (A) The expected bands at ~35 kDa and ~68 kDa were observed in RH30 cells with only endogenous PP1β (lane 3) and after transfection with PP1β-EGFP (lane 4). (B) Enhanced anti-PP1β staining was observed in RH30 cells expressing high levels of PP1β (B, arrows) after fixation with either cytoskeleton buffer + paraformaldehyde (CSK-PFA) or -20°C methanol (MeOH). Overlapping signals appear as yellow in Merges (d, h). Bars, 20 μm.

**Additional file 10: Figure S7.** Little or no signal overlap for exogenously expressed Sgcg and tensin 2 in RH30 cells. (A) Flag-tagged Sgcg, Sgcb and Sgcd were co-expressed with GFP-tagged human tensin 2. (B) Untagged Sgcg was co-expressed with Flag-tagged murine TenC1 / tensin 2. In both cases, Sgcg was stained with the Proteintech anti-Sgcg (Aa, Ae, Ba, Bd, red in merges); tensin 2 was visualized using either (A) the GFP signal (b, f, green (g) in merges) or (B) anti-Flag antibody (b, e, green in merges). (A) Actin filaments were stained with fluorescent phalloidin (c, g, blue in merges). Cells with varied expression levels are shown. Sgcg signal is associated with internal structures, as well as in peripheral punctae (arrow). Note the absence of staining in the untransfected cell in panel Aa under the arrow that points to Sgcg punctae in the transfected cell above. Both human and murine tensin 2 isoforms localize in focal adhesions at the ends of actin filaments (arrowheads), but the tensin 2-enriched spots do not co-localize with the Sgcg punctae. Bars, 20 μm.

**Additional file 11: Figure S8.** Limited signal overlap for Sgcg and MYPT2 in RH30 cells. Untagged Sgcg visualized with Proteintech anti-Sgcg (a, d, g, red in merges) was transfected with Flag-MYPT2 (b, e, h, green in merges) in RH30 cells. An occasional cell showed some overlap of Flag-MYPT2 with Sgcg at internal structures (double arrows). Most Sgcg signal was associated with internal structures and peripheral punctae (arrows) while most MYPT2 localized with phalloidin-stained actin filaments (arrowheads; phalloidin staining not shown). Signal overlaps appear as yellow-orange. Bar, 20 μm.

**Additional file 12: Figure S9.** Limited signal overlap for Sgcg and EGFP-tagged PP1β in RH30 cells. (A) Cells were co-transfected with Flag-tagged Sgcg, Sgcd, Sgcb, and the EGFP constructs shown. Sgcg was visualized with Proteintech anti-Sgcg (a, e, i; red in merges); PP1β-EGFP (b), EGFP- PP1β (f) and EGFP (j) are shown in green in merges (d, h, l). Filamentous actin was visualized with AlexaFluor350 phalloidin to show cell outlines (c,g,k; blue in merges). (B) RH30 cells transfected with Flag-tagged Sgcg, Sgcd, and Sgcb were fixed (a-d) 10 min and (e-f) 15 min after a switch to hypo-osmotic media and stained with anti-Flag antibodies (a, e; red in merges) and with the Abcam antibody against endogenous PP1β (b, f; green in merges). PP1β-associated signals were not excluded from Sgcg-associated structures, but convincing co-localizations were not observed. Nuclear DNA was visualized with DAPI (c, g; blue in merges). Bars, 20 μm.

**Additional file 13: Figure S10.** Changes in Total NKCC1 and phosphorylated (P)-NKCC1 in response to eccentric contraction (ECC) strain in WT and *Sgcg*^*-/-*^ EDL muscles. (A) Representative immunoblots. (B) The relative amount of glycosylated (170 kDa) NKCC1 tended to be lower after ECC of *Sgcg*^*-/-*^ muscles (*P* = 0.06), but amounts of the nonglycosylated (130 kDa) NKCC1 polypeptide are unchanged (*P* = 0.87). (C) Ratios of P/T-NKCC1 after ECC relative to P/T-NKCC1 at rest for WT and *Sgcg*^*-/-*^ muscles. *N* = 5-6 muscle pairs per strain. *P* = 0.07. Central bars, means; error bars, S.D. Student’s unpaired t-tests.

**Additional file 14: Figure S11.** Enlargement of the IPA diagram shown in Fig. 10.

## Data Availability

Antibody characterizations, negative data and sample reports in MS Excel format from the proteomics experiments are provided as supplementary data. The mass spectrometry proteomics data have been deposited to the ProteomeXchange Consortium via the PRIDE [124] partner repository with the dataset identifier PXD028584 and 10.6019/PXD028584. Scaffold 4.4.4 proteomics files that are the basis for Fig. 3 are available from the corresponding author, as are flash-frozen muscles from the new Svil-mutant mouse strain. Aliquots of the homemade affinity-purified anti-Sgcg and anti-mAV rabbit polyclonal antibodies also are available upon reasonable request while supplies last.

## Abbreviations

ATP: Adenosine triphosphate
Ca^++^: Calcium ions
CSK: Cytoskeletal buffer
DGC: Dystrophin glycoprotein complex
DTT: Dithiothreitol
ECC: Eccentric contractions
ECL: Enhanced chemiluminescence
ECM: Extracellular matrix
EDL: Extensor digitorum longus muscle
ERK1/2: Extracellular regulated kinases 1 and 2
GST: Glutathione s-transferase
IB: Immunoblot
IF: Immunofluorescence
IP: Immunoprecipitation
IPA: Ingenuity Pathway Analysis software
LAT1/SLC7A5: Large neutral amino acids transporter, small subunit 1
LC-MS/MS: Liquid chromatography with tandem mass spectrometry
LGMD: Limb girdle muscular dystrophy
MAPK: Mitogen-activated protein kinases
mAV: Murine archvillin
MEK1/2: MAPK/ERK kinase 1 and 2
MOPS: 3-Morpholinopropane-1-sulfonic acid
MYPT2/PPP1r12b: Myosin phosphatase target subunit 2/protein phosphatase 1, regulatory subunit 12B
NKCC1: Sodium potassium chloride co-transporter 1
PLA: Proximity ligation assay
PMSF: Phenylmethylsulfonyl fluoride
PP1β: Serine/threonine protein phosphatase catalytic subunit 1 beta
(P/T)-ERK1/2: (phospho/total) ERK1/2
RH30: SJC-RH30 human rhabdomyosarcoma cells
RIPA: Radioimmunoprecipitation assay buffer
Rx: Relaxation buffer
SLC12A2: Solute carrier family 12 member 2
SC: Sarcoglycan complex
Sgca: Alpha-sarcoglycan
Sgcb: Beta-sarcoglycan
Sgcd: Delta-sarcoglycan
Sgcg: Gamma-sarcoglycan
Svil, SV: Supervillin
TA: Tibialis anterior muscle
WCL: Whole cell lysate
