# Supplementary material for: Novel γ-sarcoglycan interactors in murine muscle membranes
Source: Skelet Muscle. 2022 Jan 22;12:2. doi: 10.1186/s13395-021-00285-2 (PMC8783446; doi:10.1186/s13395-021-00285-2)
Supplement: Supplementary file 1 — Additional file 1: Table S1. Primers used in this study. [file 13395_2021_285_MOESM1_ESM.docx]

**Additional File 1, Supplementary Table 1. Primers used in this study.**

| Primer Name | **Primer Sequence** |
| --- | --- |
| mSgcg-For-BamHI | 5’-GGATCCCATGTTACAGCAGATGGACTTC-3’ |
| mSgcg-Rev-EcoRI | 5’-GAATTCTTCAACAGACGTGGCTGTTC-3’ |
| mAV-For-BamHI | 5’-GGATCCCCAGAAGCTGACTCTGAATACTT-3’ |
| mAV-Rev-XhoI | 5’-CTCGAGCTCGGGTTGACTCCATAAA-3’ |
| Sgcg-L36Y37-BamHI-sense | 5’-GAGAAAGCGCTGT*gga*T*c*CTTGTTTGTTCTTC-3’ |
| Sgcg-L36Y37-BamHI-antisense | 5’-GAAGAACAAACAAG*g*A*tcc*ACAGCGCTTTCTC-3’ |
| Scgc-BglII-start-For | 5’- AGATCTGCTAGCATGGTGCGTGAGCAGTACACTAC-3’ |
| Scgc- EcoRI-end-Rev | 5’- GAATTCTTGTACAGCTCGTCCATGCCGAGAGT-3’ |
| Scgc- For-EcoRV-1Met | 5’- GATATCATGGTGCGTGAGCAGTACACTACAGCC-3’ |
| Scgc- Y6A-sense | 5’- GGTGCGTGAGCAG*gc*CACTACAGCCACAG-3’ |
| Scgc- Y6A-antisense | 5’- CTGTGGCTGTAGTG*gc*CTGCTCACGCACC-3’ |
| HA-CFP-Stp For | 5’-CTCTACAAGGGT*taa*CTGGAGCCGAGG-3’ |
| HA-CFP-Stp Rev | 5’-CCTGGCTCCAG*tta*ACCCTTGTAGAG-3’ |
| Guide RNA 1 | 5'-TGTAGGGCGATCCAAAGAAGAAG-3' |
| Guide RNA 2 | 5'-TCTTCAATGCTTACCTGGCTCGG-3' |

Underlined sequences, restriction sequences

*Lower case italics*, mutated bases
