# Supplementary figures and images for: Novel γ-sarcoglycan interactors in murine muscle membranes
Source: Skelet Muscle. 2022 Jan 22;12:2. doi: 10.1186/s13395-021-00285-2 (PMC8783446; doi:10.1186/s13395-021-00285-2)

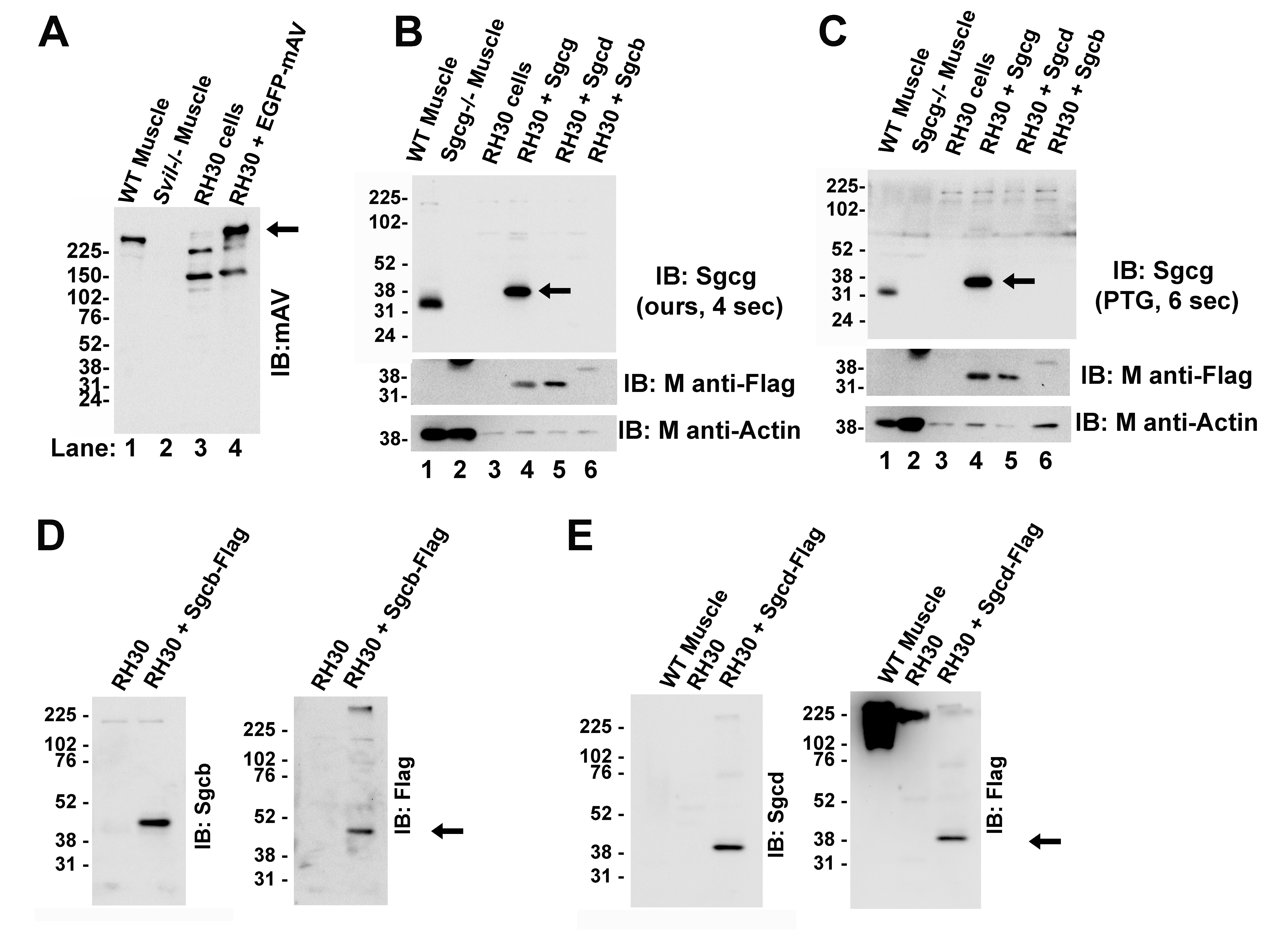

Supplement: Supplementary file 2 — Additional file 2: Figure S1. Validation of new and commercial polyclonal antibodies and expression of SC proteins in RH30 cells. New affinity-purified rabbit (Rb) antibodies were generated against murine archvillin (mAV) residues 121-568 (anti-mAV) and against Sgcg residues 72-290 (anti-Sgcg). Full-length immunoblots show that (A) anti-mAV and (B) our anti-Sgcg are specific for the expected ~245-kDa and ~35-kDa bands in mouse muscle, but extra bands are present in RH30 cell lysates. Specificity of the major bands is confirmed by their absence from muscles lacking the immunogen and by increased staining in transfected cells. (C) Staining is similar for Proteintech Group (PTG) anti-Sgcg antibody. Immunoblots of gastrocnemius muscle proteins from wild-type (WT, lanes 1) and Svil-/- or Sgcg-/- muscles (lanes 2), untransfected RH30 cells (lanes 3) and RH30 cells transfected with vectors encoding Sgcg- Flag (lanes 4), Sgcd- Flag (lanes 5) or Sgcb- Flag (lanes 6). Exogenous expression in RH30 cells was confirmed for Flag-tagged (B, C) Sgcg, (D) Sgcb and (E) Sgcd. No endogenous sarcoglycans were detected in RH30 cells. (B, C) The same blots used for anti-Sgcg staining were stripped and re-probed with anti-actin as a loading control. No cross-reactivity of anti-Sgcg was observed with the structurally similar Sgcd protein. Minor immunoreactive higher molecular mass bands at ~70 kDa and ~160 kDa in RH30 cells are variably present. These larger bands may represent SDS-resistant complexes of sarcoglycans [49, 63]. Positions of molecular mass markers in kDa are shown on the left. Arrows denote specific bands of the expected size. [file 13395_2021_285_MOESM2_ESM.tif]

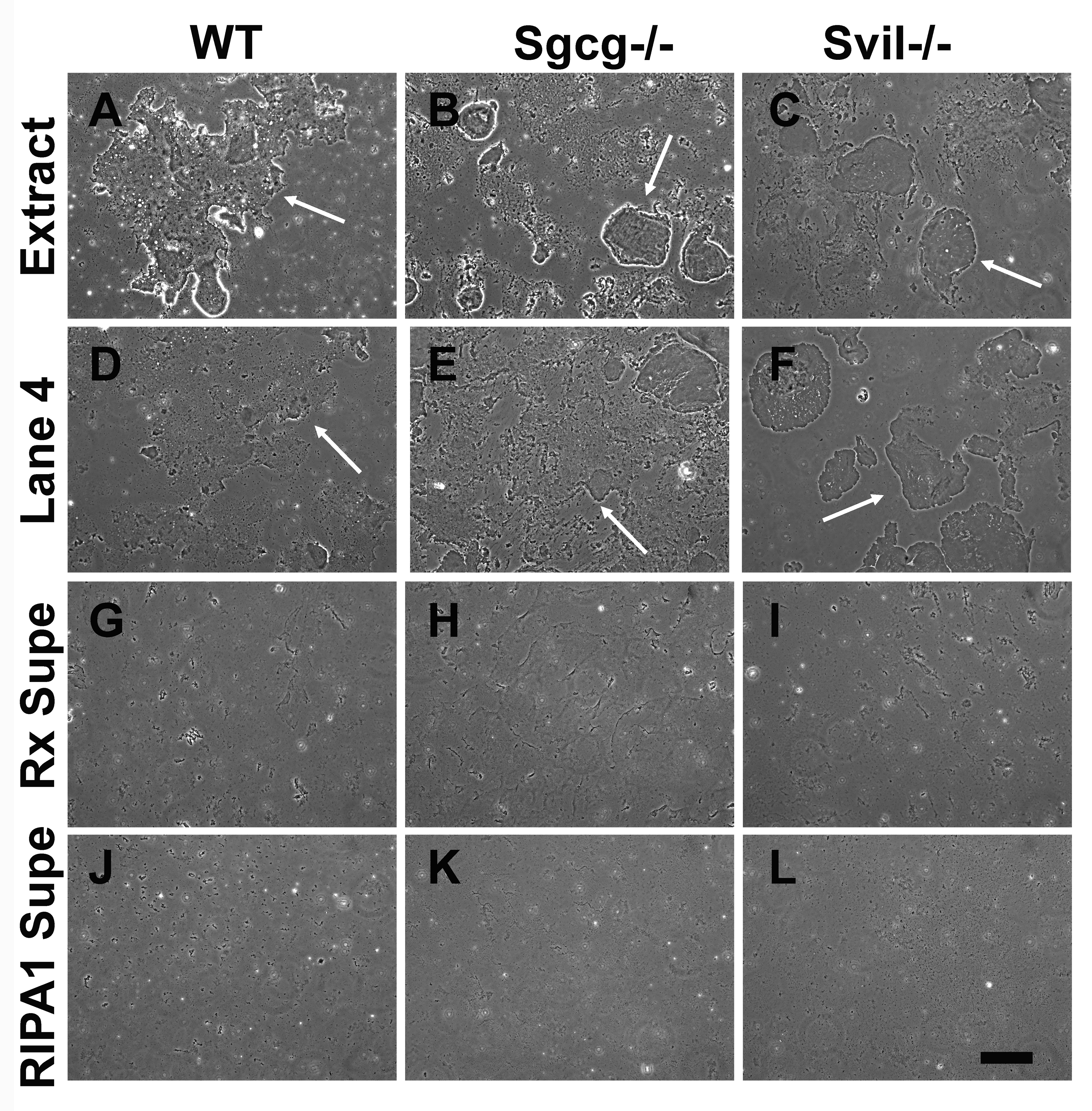

Supplement: Supplementary file 5 — Additional file 5: Figure S2. Phase contrast micrographs of gastrocnemius muscle membrane fractions. Fractions, referenced as described in Fig. 1A, from (A, D, G, J) C57BL/6 (WT), (B, E, H, K) Sgcg-/- and (C, F, I, L) Svil-/- mice were visualized by phase-contrast microscopy. Large membrane fragments (arrows) were observed in (A-C) total muscle extracts and (D-F) crude membrane pellets (Lane 4), but only small particulates were present in the supernatants after extraction with (G-I) Rx or (J-L) RIPA1 buffers. Bar, 0.2 mm. [file 13395_2021_285_MOESM5_ESM.tif]

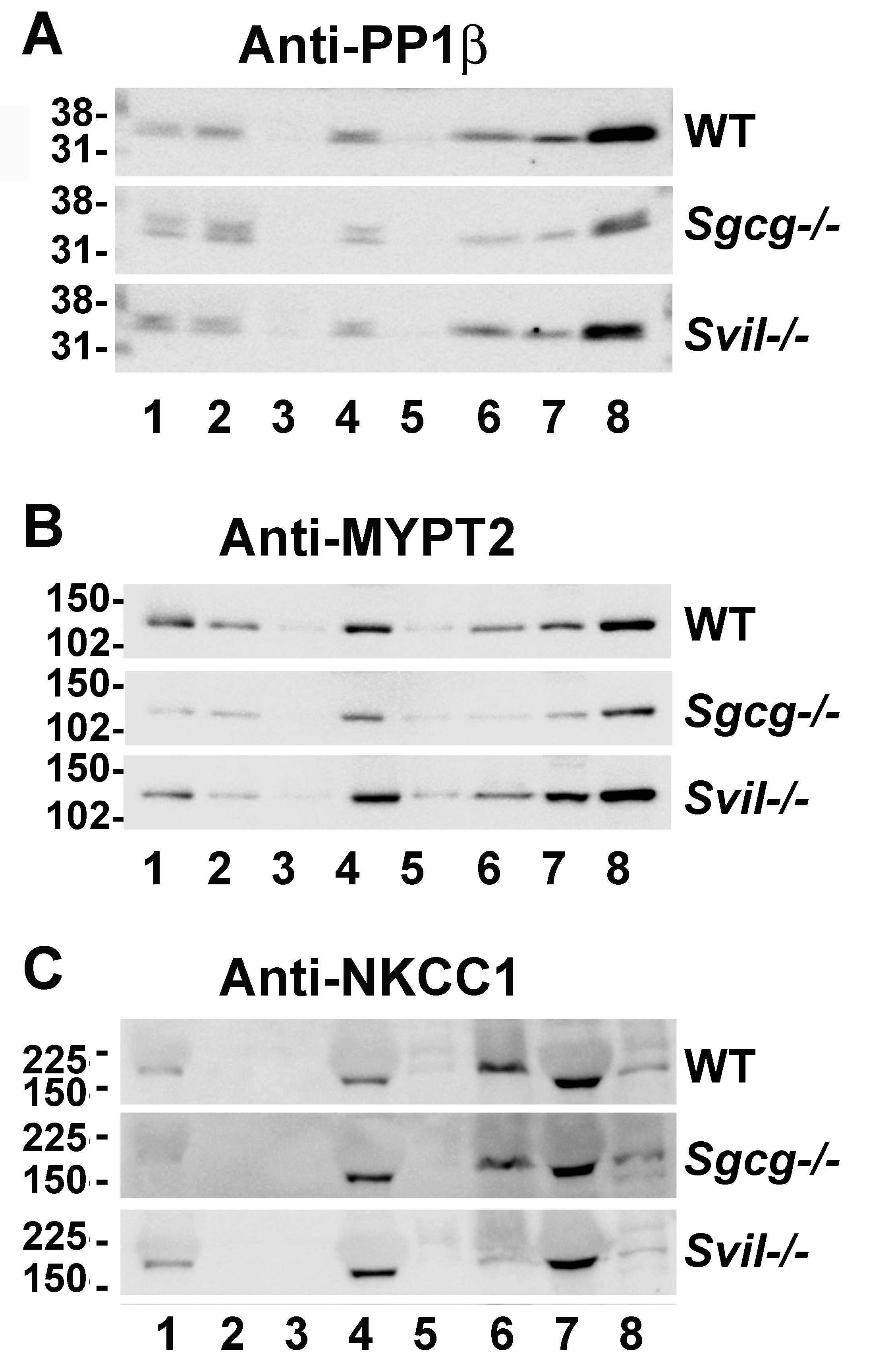

Supplement: Supplementary file 6 — Additional file 6: Figure S3. Fractionation of selected candidate Sgcg interactors from the different muscle genotypes. Fractions from ~100 mg skeletal muscle were extracted as shown in Fig. 1, immunoblotted and probed for the Sgcg candidate interactors PP1β (Life Span Biosciences), MYPT2 (Proteintech Group), or NKCC1 (Alomone Laboratories). All fractions are normalized as in Fig. 2. Each immunoblot is representative of 3 (Sgcg-/-, Svil-/-) or 4 (WT) independent biological replicates. The multiple bands for NKCC1 are a consequence of differential glycosylation [74, 75]. [file 13395_2021_285_MOESM6_ESM.tif]

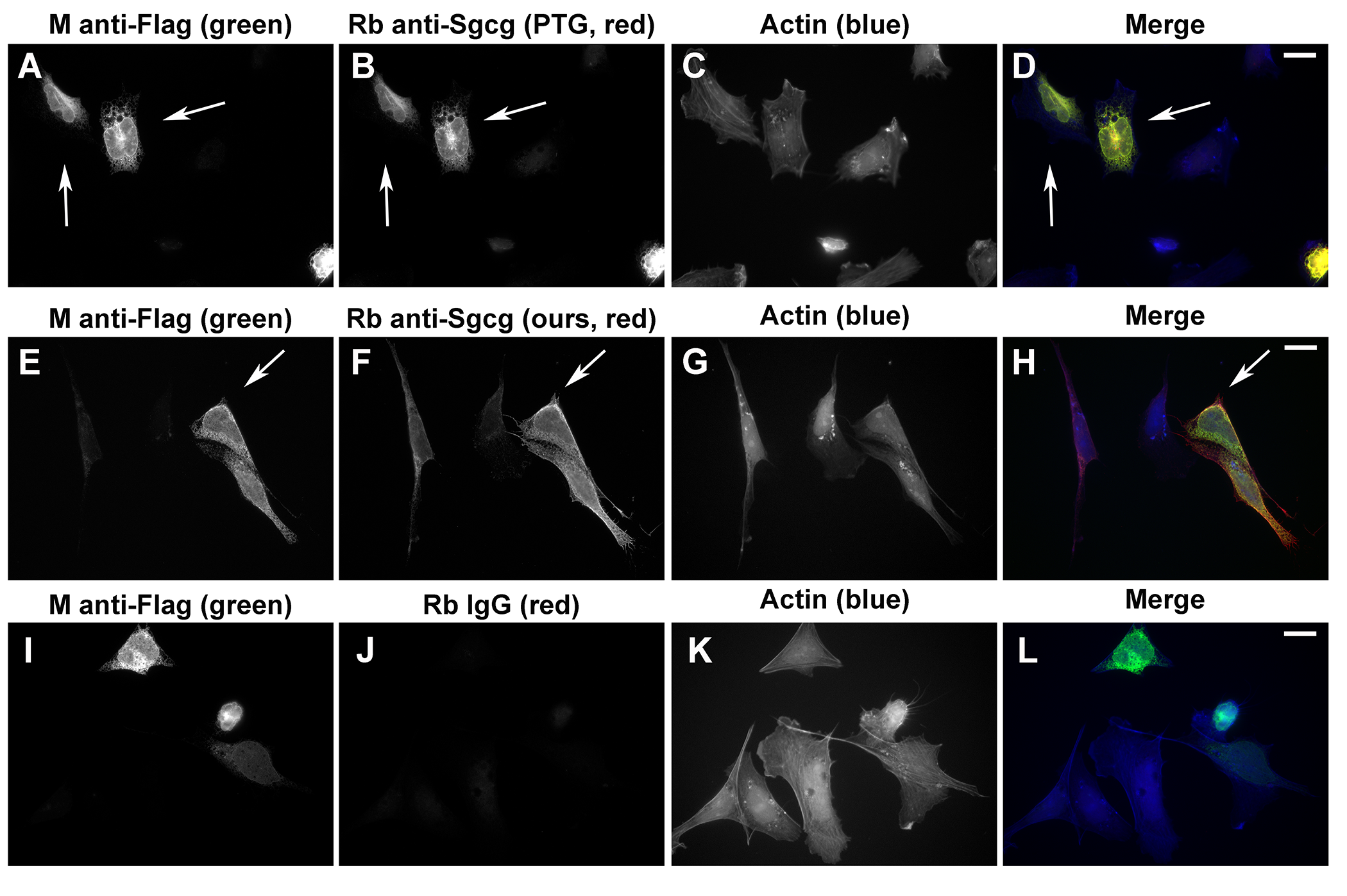

Supplement: Supplementary file 7 — Additional file 7: Figure S4. Validation of rabbit (Rb) polyclonal antibodies against Sgcg for immunofluorescence microscopy. Evaluation of Proteintech Group Inc. (PTG, A-D) and our new affinity-purified (ours, E-H) anti-Sgcg antibodies for immunofluorescence microscopy, as compared to nonspecific Rb IgG (I-L), in RH30 cells transfected with plasmids encoding Sgcg-Flag. Both anti-Sgcg antibodies specifically recognize structures stained with anti-Flag (arrows). Overlapping red and green signals appear as yellow. [file 13395_2021_285_MOESM7_ESM.tif]

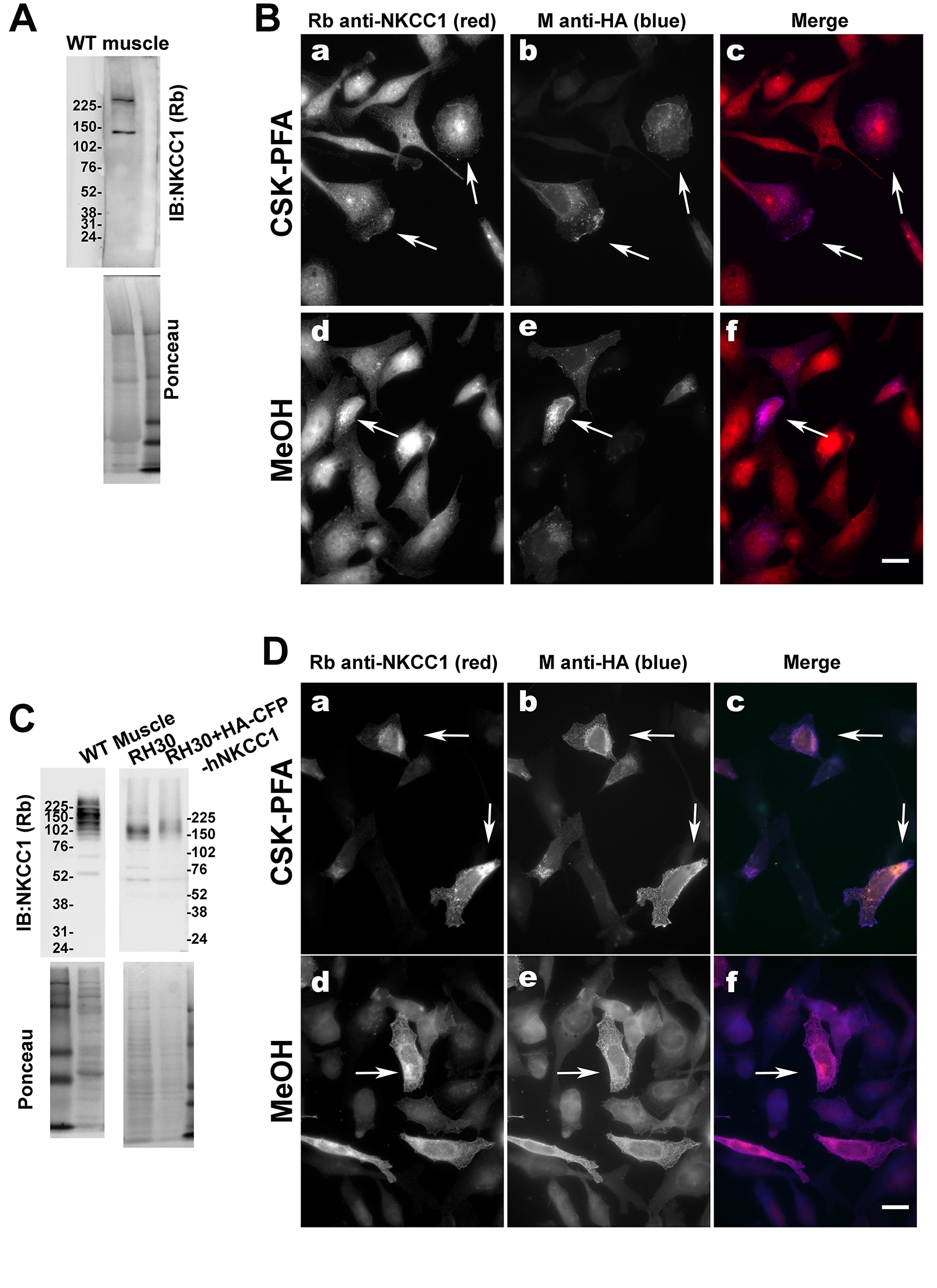

Supplement: Supplementary file 8 — Additional file 8: Figure S5. Validation of rabbit (Rb) polyclonal antibodies against NKCC1. (A, C) Full-length immunoblots of mouse muscle and RH30 cells with and without transfected HA-CFP-NKCC1; (B, D) Anti-NKCC1 (panels a, d; red in merges) and murine (M) anti-HA staining (panels b, e; blue in merges) in RH30 cells transfected with HA-CFP-NKCC1. Anti-NKCC1 antibodies were from (A, B) Alomone Labs, #ANT-071 and (C, D) Proteintech Group, #13884-1-AP. Both anti-NKCC1 antibodies recognized the expected bands in the 97-220 kDa molecular mass range (A, C). Similar staining patterns have been reported previously, with the multiplicity of bands attributed to differential splicing and variations in glycosylation [74, 75]. In immunofluorescence (B, D), only the Proteintech antibody (Da, Dd) exhibited enhanced staining in RH30 cells expressing high levels of exogenous HA-CFP-NKCC1 (B, D, arrows). Overlapping signals appear as magenta in Merges (c and f). Bars, 20 μm. [file 13395_2021_285_MOESM8_ESM.tif]

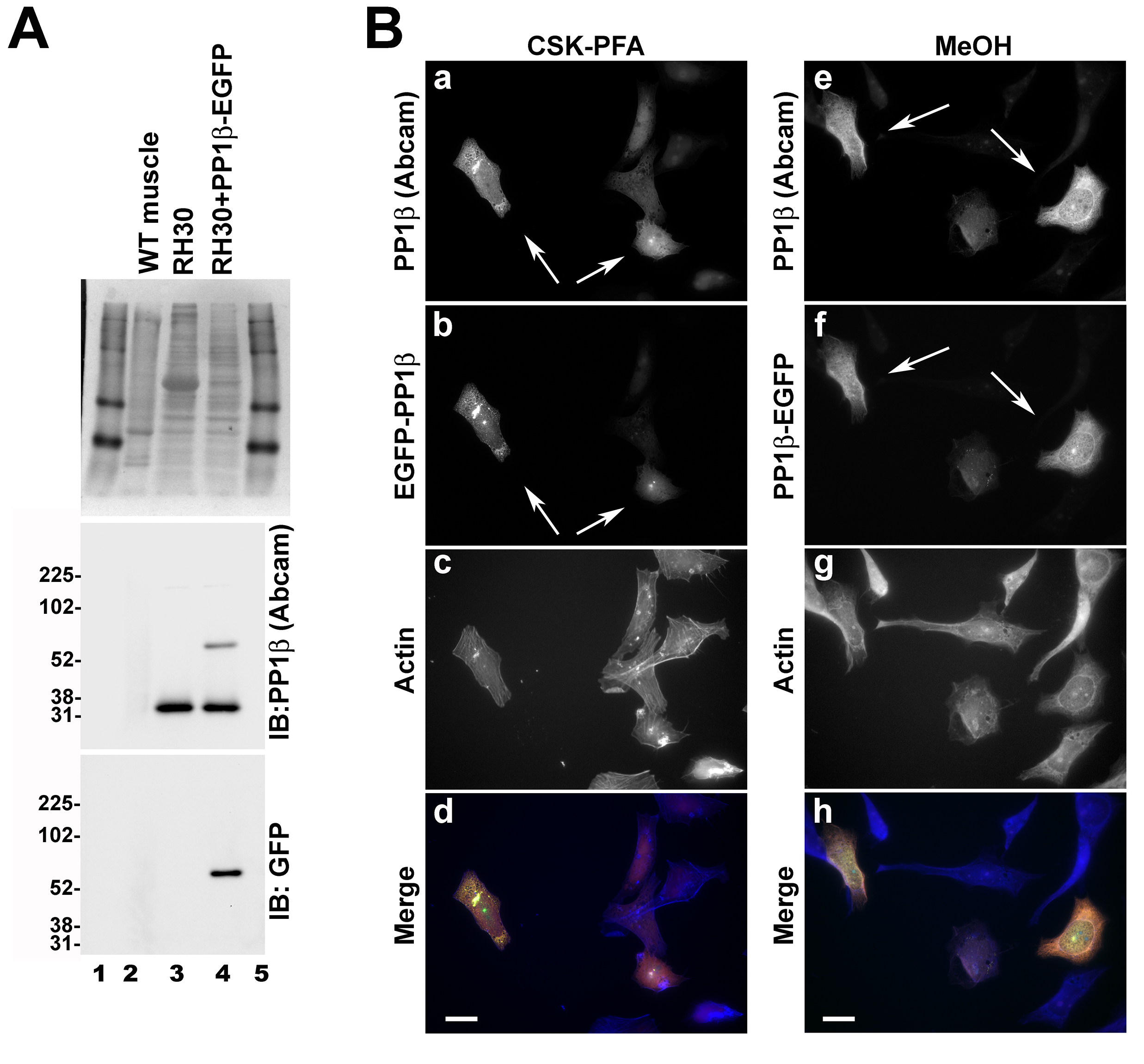

Supplement: Supplementary file 9 — Additional file 9: Figure S6. IF signal of AbCam rabbit monoclonal anti- PP1β correlates with EGFP signal from EGFP-PP1β or PP1β-EGFP. (A) Full-length immunoblots of mouse muscle and RH30 cells with and without transfected PP1β-EGFP, as indicated; lanes 1 and 5 show molecular mass markers. (B) Anti-PP1β (panels a, e; red in merges) and EGFP fluorescence (panels b, f; green in merges) in RH30 cells transfected with PP1β cDNA, as indicated. (A) The expected bands at ~35 kDa and ~68 kDa were observed in RH30 cells with only endogenous PP1β (lane 3) and after transfection with PP1β-EGFP (lane 4). (B) Enhanced anti-PP1β staining was observed in RH30 cells expressing high levels of PP1β (B, arrows) after fixation with either cytoskeleton buffer + paraformaldehyde (CSK-PFA) or -20°C methanol (MeOH). Overlapping signals appear as yellow in Merges (d, h). Bars, 20 μm. [file 13395_2021_285_MOESM9_ESM.tif]

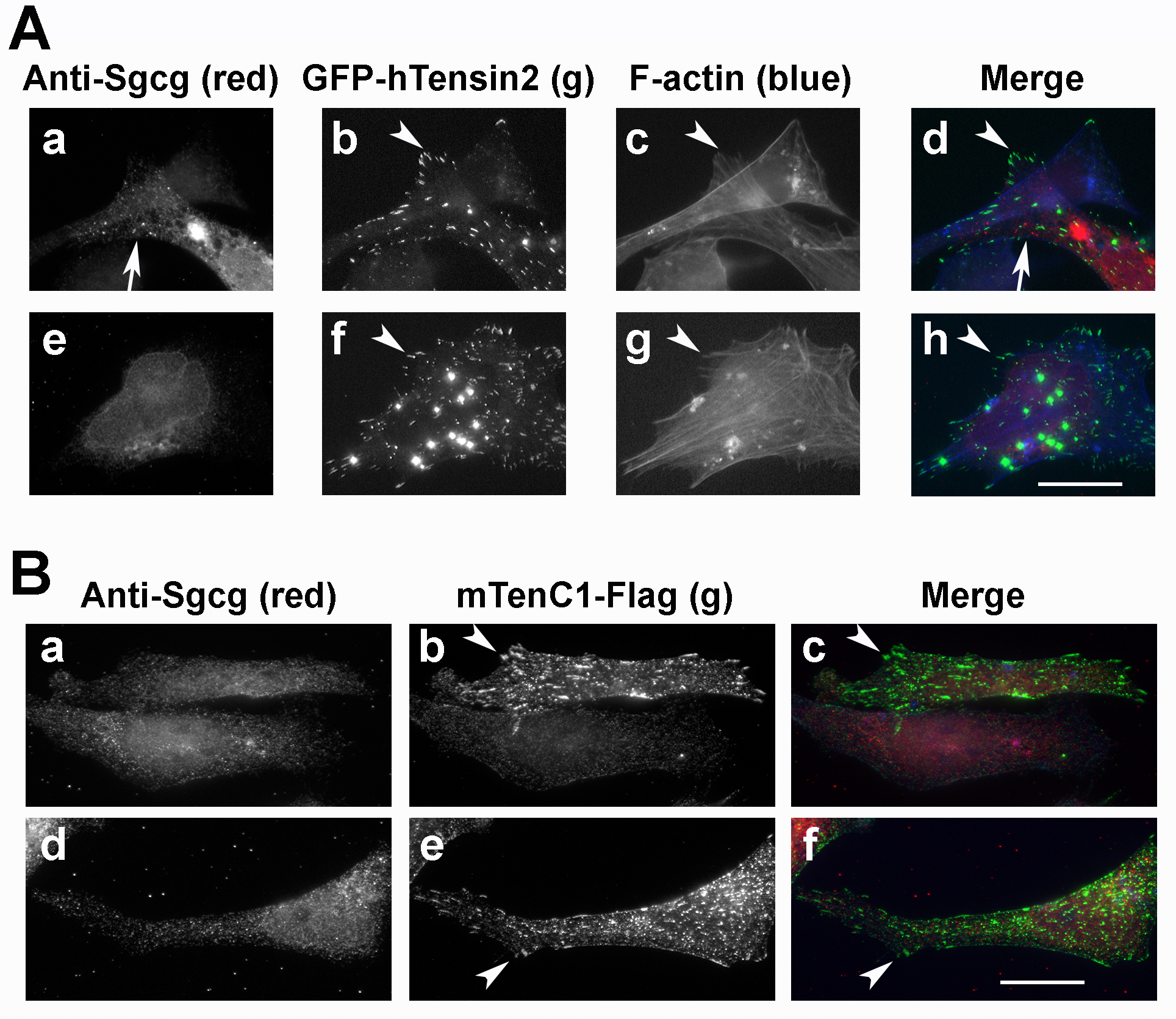

Supplement: Supplementary file 10 — Additional file 10: Figure S7. Little or no signal overlap for exogenously expressed Sgcg and tensin 2 in RH30 cells. (A) Flag-tagged Sgcg, Sgcb and Sgcd were co-expressed with GFP-tagged human tensin 2. (B) Untagged Sgcg was co-expressed with Flag-tagged murine TenC1 / tensin 2. In both cases, Sgcg was stained with the Proteintech anti-Sgcg (Aa, Ae, Ba, Bd, red in merges); tensin 2 was visualized using either (A) the GFP signal (b, f, green (g) in merges) or (B) anti-Flag antibody (b, e, green in merges). (A) Actin filaments were stained with fluorescent phalloidin (c, g, blue in merges). Cells with varied expression levels are shown. Sgcg signal is associated with internal structures, as well as in peripheral punctae (arrow). Note the absence of staining in the untransfected cell in panel Aa under the arrow that points to Sgcg punctae in the transfected cell above. Both human and murine tensin 2 isoforms localize in focal adhesions at the ends of actin filaments (arrowheads), but the tensin 2-enriched spots do not co-localize with the Sgcg punctae. Bars, 20 μm. [file 13395_2021_285_MOESM10_ESM.tif]

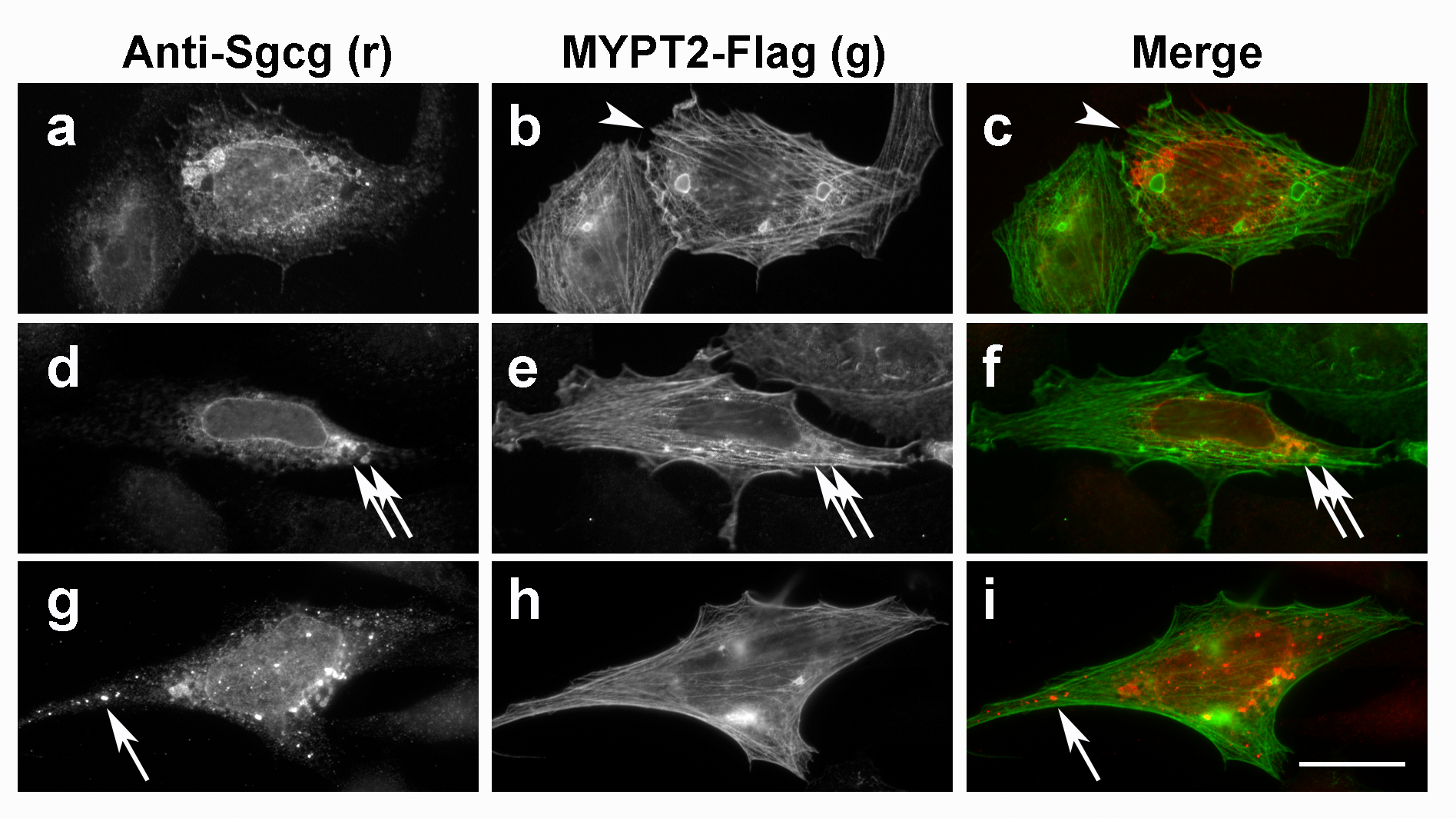

Supplement: Supplementary file 11 — Additional file 11: Figure S8. Limited signal overlap for Sgcg and MYPT2 in RH30 cells. Untagged Sgcg visualized with Proteintech anti-Sgcg (a, d, g, red in merges) was transfected with Flag-MYPT2 (b, e, h, green in merges) in RH30 cells. An occasional cell showed some overlap of Flag-MYPT2 with Sgcg at internal structures (double arrows). Most Sgcg signal was associated with internal structures and peripheral punctae (arrows) while most MYPT2 localized with phalloidin-stained actin filaments (arrowheads; phalloidin staining not shown). Signal overlaps appear as yellow-orange. Bar, 20 μm. [file 13395_2021_285_MOESM11_ESM.tif]

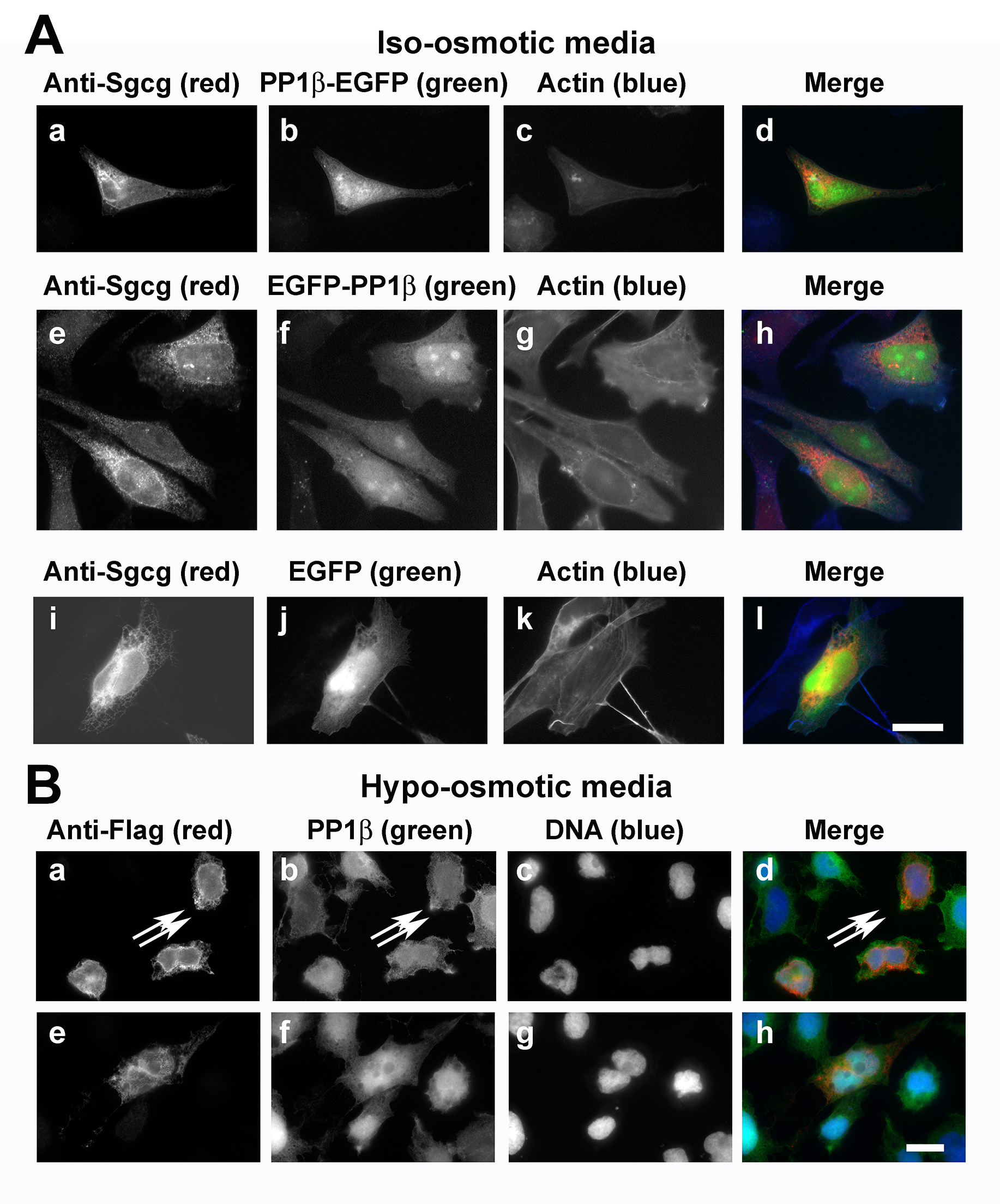

Supplement: Supplementary file 12 — Additional file 12: Figure S9. Limited signal overlap for Sgcg and EGFP-tagged PP1β in RH30 cells. (A) Cells were co-transfected with Flag-tagged Sgcg, Sgcd, Sgcb, and the EGFP constructs shown. Sgcg was visualized with Proteintech anti-Sgcg (a, e, i; red in merges); PP1β-EGFP (b), EGFP- PP1β (f) and EGFP (j) are shown in green in merges (d, h, l). Filamentous actin was visualized with AlexaFluor350 phalloidin to show cell outlines (c,g,k; blue in merges). (B) RH30 cells transfected with Flag-tagged Sgcg, Sgcd, and Sgcb were fixed (a-d) 10 min and (e-f) 15 min after a switch to hypo-osmotic media and stained with anti-Flag antibodies (a, e; red in merges) and with the Abcam antibody against endogenous PP1β (b, f; green in merges). PP1β-associated signals were not excluded from Sgcg-associated structures, but convincing co-localizations were not observed. Nuclear DNA was visualized with DAPI (c, g; blue in merges). Bars, 20 μm. [file 13395_2021_285_MOESM12_ESM.tif]

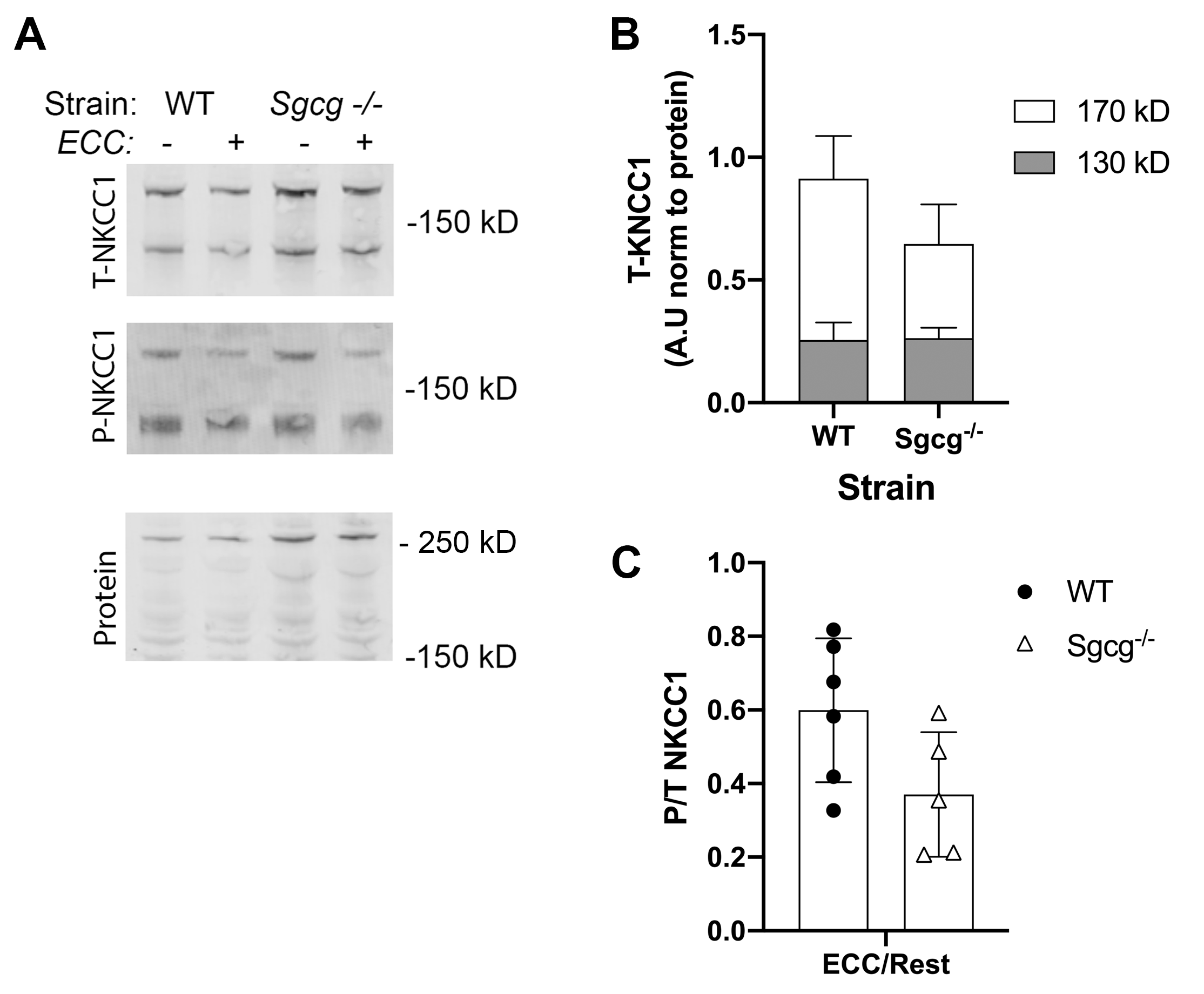

Supplement: Supplementary file 13 — Additional file 13: Figure S10. Changes in Total NKCC1 and phosphorylated (P)-NKCC1 in response to eccentric contraction (ECC) strain in WT and Sgcg-/- EDL muscles. (A) Representative immunoblots. (B) The relative amount of glycosylated (170 kDa) NKCC1 tended to be lower after ECC of Sgcg-/- muscles (P = 0.06), but amounts of the nonglycosylated (130 kDa) NKCC1 polypeptide are unchanged (P = 0.87). (C) Ratios of P/T-NKCC1 after ECC relative to P/T-NKCC1 at rest for WT and Sgcg-/- muscles. N = 5-6 muscle pairs per strain. P = 0.07. Central bars, means; error bars, S.D. Student’s unpaired t-tests. [file 13395_2021_285_MOESM13_ESM.tif]

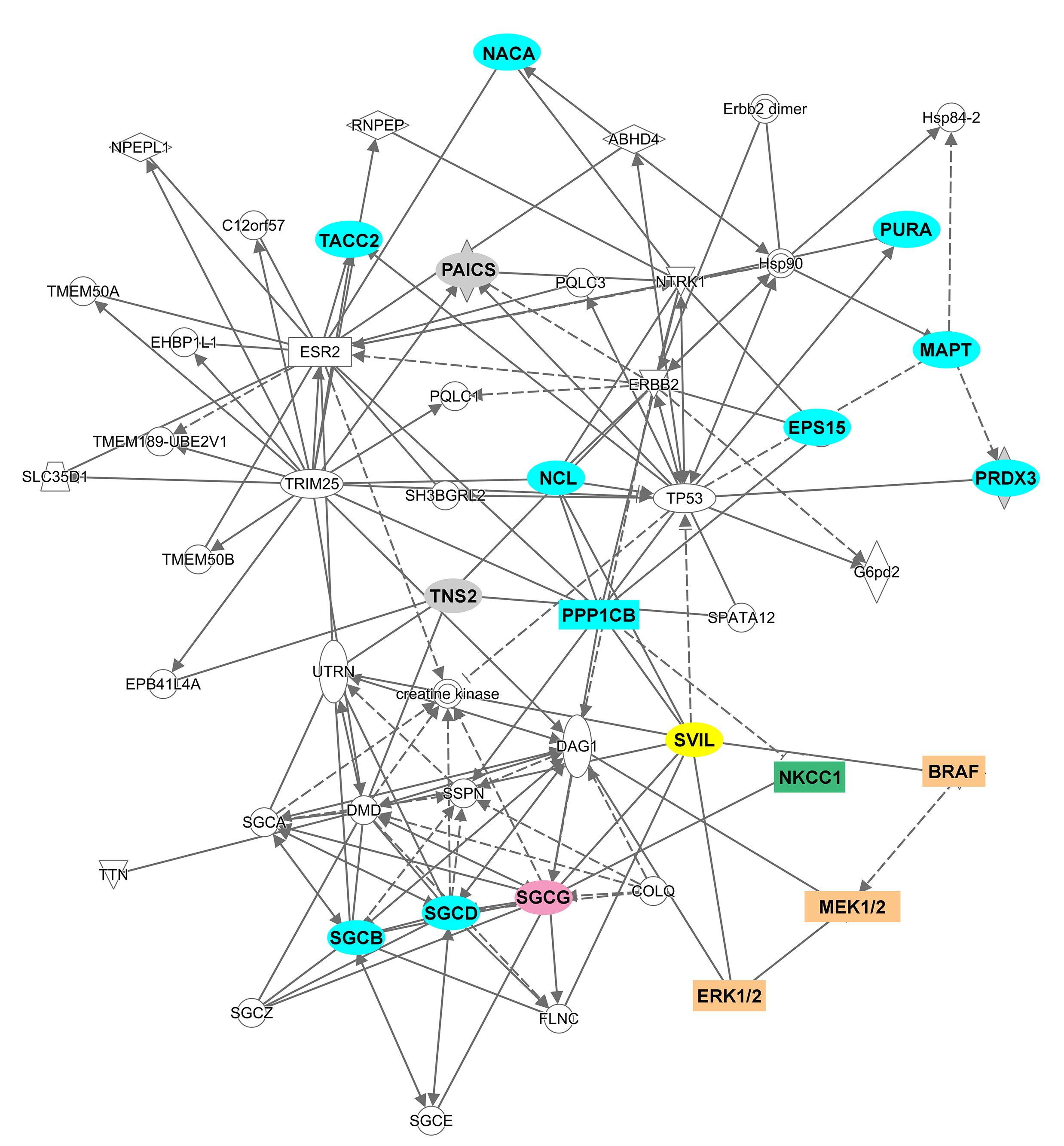

Supplement: Supplementary file 14 — Additional file 14: Figure S11. Enlargement of the IPA diagram shown in Fig. 10. [file 13395_2021_285_MOESM14_ESM.tif]
